# Development and validation of the AI-predictive ParaScout *in-vitro* diagnostic (IVD) system for the microscopic detection of gastro-intestinal helminths in stool

**DOI:** 10.1080/22221751.2026.2698240

**Published:** 2026-07-01

**Authors:** Ihor Feoktistov, Rob Koelewijn, Chris Tieken, Lisette van Lieshout, Serena Slavenburg, Rens Zonneveld, Jaap J. van Hellemond

**Affiliations:** a Leven Vision, Leiden, The Netherlands; b Department of Medical Microbiology and Infectious Diseases, Erasmus MC University Medical Center, Rotterdam, The Netherlands; c Parasitology Research Group, Leiden University Center for Infectious Diseases (LUCID), Leiden University Medical Center (LUMC), Leiden, The Netherlands; d Regional Public Health Laboratory Kennemerland, Haarlem, The Netherlands; e Department of Medical Microbiology and Infection Prevention, Amsterdam UMC, Amsterdam, The Netherlands

**Keywords:** Artificial intelligence (AI), machine learning, diagnostic microbiology, gastro-intestinal helminths, clinical parasitology, microscopy

## Abstract

The comprehensive method to detect gastro-intestinal infections is still microscopic examination of stool specimens. However, manual microscopic examination is laborious, and its accuracy is highly observer-dependent. Therefore, we have developed a deep-learning approach integrated with a digital microscope scanner to detect gastro-intestinal helminths in digital images, which allows faster and more precise detection of gastro-intestinal helminths. A novel *in-vitro* diagnostic (IVD) system (ParaScout) was developed that consists of an affordable, commercially available microscope scanner and a cloud-based deep-learning algorithm for accurate identification of gastro-intestinal helminths in stool. The performance of the ParaScout system was compared to manual examination by 4 expert technicians, using 50 validated stool specimens containing either no gastro-intestinal helminths or one or more of the 15 gastro-intestinal helminth species for which the ParaScout system had been trained. In the 45 positive stool specimens, 63 gastro-intestinal helminth species were present, and therefore, examination by 4 technicians could have revealed 252 gastro-intestinal helminth identifications (4*63). In total, the manual examinations produced 16 false negative and 11 false positive results, resulting in a sensitivity of 93.7% and a specificity of 99.6%. When ParaScout was employed to highlight suspected structures for human expert confirmation, these values increased to 98.8% and 99.9%, respectively. This study demonstrated that automated scanning in combination with a deep-learning algorithm can detect gastro-intestinal helminths in stool samples with high sensitivity, while resulting in minimal false positive detections if combined with expert confirmation. This approach shows high promise for automating and improving the quality of gastro-intestinal helminth detection in patient diagnostics.

## Introduction

Gastro-intestinal helminths are among the most common infections worldwide, with soil-transmitted helminths infecting an estimated 1.5 billion people [1]. Although some gastro-intestinal helminths can cause severe disease, e.g. *Strongyloides stercoralis*, helminthiasis are still neglected diseases for which improved diagnostic methods are required [1]. The classical method to detect gastro-intestinal infections is microscopic examination of stool specimens [2]. However, this all-round method that allows detection of all gastro-intestinal helminths is laborious, and its accuracy is highly observer-dependent [3]. Nucleic acid amplification techniques (NAATs) are recognised for their superior sensitivity and specificity in diagnostics [4]. However, due to the extensive diversity of gastro-intestinal helminths infecting humans – over 15 species, including several zoonotic types spanning nematodes, cestodes, and trematodes – a universal NAAT capable of detecting all these species is not commercially available, and if developed, it will be too costly for routine diagnostics. Additionally, parasite identification is often about ruling out mimics; for instance, a mucus strand from a true helminth, which cannot be done by NAAT. For these reasons, there is still a need for a reliable all-round detection method for gastro-intestinal helminths in stool.

Automated microscopic scanning, in combination with deep-learning-based object detection, is a powerful technology that could relieve the drawbacks of microscopic detection of parasites [5]. Multiple examples of such an application exist for the microscopic detection of malaria parasites in whole blood [6,7], and machine learning models were shown to be able to detect helminth eggs in images of stool [8–11]. In addition, several studies have evaluated the performance of more comprehensive automated AI-predictive IVD systems for the detection of multiple gastro-intestinal helminths in stool. These studies were either focused on affordable mobile devices for use in endemic and resource-limited settings to detect predominantly soil-transmitted helminths and *Schistosoma* [12,13], or fully automated and/or expensive microscope scanners for use in high-income countries to detect gastro-intestinal helminths as optimally as possible [14,15]. In this study, a novel *in-vitro* diagnostic (IVD) system, ParaScout, was developed in collaboration with Leven Vision (https://www.leven.vision/) (Leiden, the Netherlands). The ParaScout IVD consists of a relatively affordable, commercially available microscope scanner and a cloud-based deep-learning algorithm for accurate identification of helminths in stool in high-income countries. The performance of this IVD was evaluated by comparing the results of manual examination by experts of multiple Dutch expertise centres for clinical parasitology with those of the AI-predictive ParaScout examination.

## Methods

For the microscopic detection of gastro-intestinal helminths in stool, Leven Vision has developed the AI-predictive IVD system called ParaScout. The development of ParaScout is described in supplementary material 1, and its functioning is shown in Figure 1 and a video (supplementary material 2). In short, the commercially available Grundium Ocus 40 automated microscope scanner (Tampere, Finland) was used to prepare an extensive data set comprising 92,757 annotated images of a very large number of well-defined stool specimens collected over a 20-year period from multiple sources and comprising 15 distinct helminth species (supplementary material 3). Helminth eggs and/or larvae were manually annotated, and subsequently, deep-learning approaches were used to develop an automated object detection model to identify helminths, resulting in object selection combined with a confidence score.

**Figure 1.**
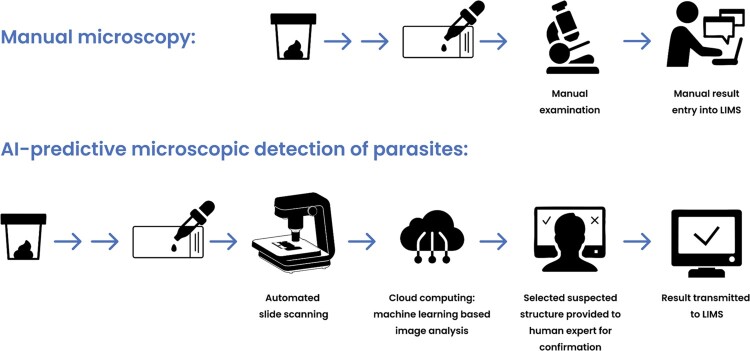
Schematic representation of the experimental procedure of manual and AI-predictive microscopic detection of gastro-intestinal helminths in stool.

The neural network model version 5.4.8 of ParaScout was used to compare the performance of the AI-predictive ParaScout IVD system in combination with human expert confirmation to expert manual examination for a set of 50 well-validated formalin-preserved stool specimens that were a minor part of the stool samples used for training. These specimens consisted of 16 remaining materials of EQA samples, 8 stool samples spiked with purified helminth eggs, and 26 derived from the Leiden University Medical Center (LUMC) or Erasmus University Medical Center educational training set. Samples were collected in distinct parts of the world (Europe, Asia, and Africa), and the set of samples comprised 13 of the 15 different helminth species for which the IVD system had been trained. Wet mount specimens for microscopic examination were prepared in a single well on 3-well-printed microscope slides (14 mm, Epredia, Michigan, USA) with a 22 × 22 mm coverslip sealed with nail polish. Each slide was scanned by the ParaScout system in ca. 6 min. The full processing sequence, which includes scanning, data export, upload, and AI interpretation, requires ca. 12 min in total, although these operations run concurrently. As a result, the system can begin scanning a new slide every ca. 6 min while export (ca. 5 min), upload (ca. 20 s), and interpretation (ca. 40 s) of the previous slide proceed in parallel. Prepared slides contained either none (n = 5, 10% of total) or one or multiple gastro-intestinal helminth species (n = 45, 90% of total, see supplementary material 4). The intended results were blinded to all examiners and defined as follows: (1) for remaining material of EQA samples, the intended result by the EQA provider was used as the gold standard, (2) for negative stool samples that were spiked with purified eggs of certain helminth species, the spiked species was used as the gold standard, and (3) for samples of the Erasmus MC and LUMC stool collection that are used for training and internal quality control purposes, the predefined presence of helminth species was used. Since this set of stool specimens has very often been examined for both training and internal quality control purposes, the presence and absence of helminth species were well defined. In case of discrepant results, images and slides were re-examined by expert manual microscopy. Molecular testing of the included stool samples was not performed because all samples were stored in formalin, which prohibits proper nucleic acid amplification techniques.

All slides were scanned by the ParaScout system before and after the manual microscopic examination by four expert technicians from three distinct centres of expertise for clinical parasitology in the Netherlands: Amsterdam UMC, Erasmus MC, and Regional Public Health Laboratory Kennemerland, Haarlem. Technicians scored the number of observed gastro-intestinal helminth eggs and larvae for each gastro-intestinal helminth species that were present. Fully automated image interpretation by the ParaScout system was used to confirm the probability threshold at which qualitative false-negative results were excluded. This threshold was subsequently used for the AI-predictive mode, where all selected structures with a probability score above the set threshold were digitally presented to four human expert technicians from the three distinct expertise centres for clinical parasitology in the Netherlands. These expert technicians then digitally examined the selected structures to confirm or reject them as a specific gastro-intestinal helminth species. In case the digital images were not sufficiently sharp for reliable interpretation, the technician could choose the option “needs manual re-examination”.

Sensitivity and specificity were calculated per species per specimen. Hence, each specimen was evaluated separately for each parasite species. Each “specimen × species” pair is thus an independent binary classification event, resulting in (50 × 15) 750 result pairs. Statistical analysis using the appropriate and common formulas was performed in Google Sheets.

## Results

First, the stability of the prepared wet mount stool slides was investigated by comparison of the detected gastro-intestinal helminth species by automated ParaScout scan before and after the manual examination. The qualitative outcome for all 50 slides was identical and only some minor differences in the quantity of detected gastro-intestinal helminth eggs were observed (on average 4 ± 10 eggs per slide, supplementary material 5). Minor quantitative differences were expected as handling of wet mount slides will cause some movement of the structures within the sealed slides, which could result in hidden or unhidden gastro-intestinal helminths. Detailed analysis of the observed small discrepancies showed that more gastro-intestinal helminths were detected in the scans prepared after manual examination compared to before manual examination, and that the most likely cause for this change is the sedimentation of structures in the freshly prepared slides was not entirely completed when the first scan was collected (see supplementary material 5). Hence, these results demonstrated the stability of the prepared wet mount stool slides throughout the entire examination procedure, and therefore, a valid performance comparison can be made between manual and ParaScout evaluation.

In a single day, 4 technicians each examined 50 slides with a surface of 1.54 × 10 mm^2^. In these 50 slides, 63 gastro-intestinal helminth species were present (range number of eggs and larvae per slide varied from 1 to >200), and therefore, the intended total number of positive results was 252 (4 technicians x 63 gastro-intestinal helminth species). In total, the 4 technicians reported 16 false negative results for 252 intended positive results: *Capillaria* spp. (3 out of 4), hookworm (4 out of 40), *Schistosoma japonicum* (1 out of 12), *Strongyloides stercoralis* (5 out of 16), and *Taenia* spp. (3 out of 40). In addition, 11 false positive results were reported: *Diphyllobothrium* spp. (1x), *Fasciola* (1x), hookworm (2x), *S. haematobium* (1x), *S. japonicum* (1x), *S. mansoni* (1x), *Taenia* spp. (1x), and *Trichuris trichiura* (3x). Most of the false results were caused by incorrect species determination: e.g. *Fasciola* as *Diphyllobothrium* (1x), *S. japonicum* as hookworm (2x), and *Capillaria* as *T. trichiura* (3x). Therefore, expert manual examination resulted in several false negative results as well as some false positive identifications (sensitivity 93.65% and specificity of 99.60%, Table 1).

**Table 1.** Performance of manual microscopy and AI-predictive ParaScout examination of stool specimens for the detection of gastro-intestinal helminths.

Examination type	Number of results				Sensitivity	Specificity
	False neg.	False pos.	True neg.	True pos.	(%)	(%)
Manual microscopy	16	11	2737	236	93.65	99.60
AI-predictive ParaScout	3	4	2744	249	98.81	99.85

Full automatic examination by the ParaScout system also resulted in either false negatives or false positive outcomes depending on the chosen threshold (supplementary material 4 and 6). Analysis of the incorrect identifications (supplementary material 7) showed that most mistakes were caused by the mix-up of hookworm eggs and *Ascaris* spp. eggs without a protein surface, incorrect *Schistosoma* species determination (spine not always visible), artefacts identified as *S. stercoralis* larvae, and a few others (see supplementary material 7).

In the AI-predictive mode, where ParaScout highlights suspected structures above the threshold for human expert confirmation, performance was nearly perfect: achieving a sensitivity of 98.8% and a specificity of 99.9% (Table 1). Using the predefined probability threshold of 0.6 the ParaScout system also detected in this study all intended gastro-intestinal helminth species in all specimens, and confirmation by human expert technicians resulted in nearly perfect results: only 3 false negative results (3x a single *H. diminuta* egg in a specimen was classified as an artefact, probably due to too blurry images) and 4 false positive results, of which 1 was probably caused by “mis-clicking” from the gastro-intestinal helminth species list (see also supplementary material 7). In 10.7% (range 4.7–15.9%) of the identified objects of a suspected gastro-intestinal helminth group in positive specimens, the image sharpness was insufficient for digital confirmation with certainty (see supplementary material 8). In these cases, manual microscopic examination is recommended.

## Discussion

The combination of human expert technicians with the selected suspected structures by the ParaScout system proved to be the superior methodology for the detection of gastro-intestinal helminths in stool with a sensitivity of 98.8% and a specificity of 99.9%. Thus, the ParaScout IVD system is so far the most affordable IVD system that has been evaluated with such high accuracy [14,15]. Furthermore, this study confirmed that even highly skilled technicians make mistakes in manual microscopic examination. Some of these mistakes were probably caused because the manual microscopy had to be performed with identical microscopes that lacked a length measurement tool in the ocular lens. In addition, all false negative results were reported for the low-abundant gastro-intestinal helminth species (<5 eggs or larvae) present in the 13 specimens that comprised two or more gastro-intestinal helminth species. Nevertheless, these results confirm the expert opinion that the accuracy of manual microscopic stool examination is limited, even for top expert technicians [2].

Fully automated ParaScout examination using the algorithm 5.4.8 version also resulted in false results. These errors, mainly caused by a misclassification of *Schistosoma* species eggs, hookworm and *Ascaris* eggs, or the identification of artefacts as *S. stercoralis* larvae, can easily be reduced by further training of the algorithm (e.g. by extension of the data-set for these cases). In circa 10% of positive cases, manual microscopic examination was required for confirmation of the gastro-intestinal helminth species (supplementary material 9). With a positivity rate in high-income countries for gastro-intestinal helminths in stool of only a few per cent of samples, manual confirmation in 10% of those cases would result in manual examination of less than 1% of the total number of examined clinical samples. In addition, microscopic examination for the detection of gastro-intestinal helminths is more difficult in slides prepared by a direct faecal smear, as was used in this study, than it is for slides prepared with stools after concentration techniques. The sediments of these concentration techniques contain only fine structures because the large structures have been removed, and therefore, these slides are thinner, and objects will be positioned next to each other instead of on top of each other. Hence, confirmation by manual microscopic examination is expected to be required only incidentally in high-income countries.

In addition to the ParaScout system, a few other systems have been developed for the automated microscopic detection of a comprehensive set of helminths in human stool. These systems can be divided into two types: systems that include fully automated stool sample handling (the KU-F40 system developed by Zhuhai Keyu Biological Engineering Co., Ltd., Guangdong, China, and the FA280 system developed by Orienter, Chengdu, Sichuan, People’s Republic of China) [15-17], and systems that only automatically examine pre-prepared stool slides (ParaScout and the system developed by Techcyte Inc., Orem, UT) [14]. Each setup has its own advantages and disadvantages. The fully automated systems of course save the manual handling of stool specimens, and therefore, allow very efficient processing and high-throughput analysis. However, automated stool processing for microscopic analysis by the so far developed systems results in diluted suspensions compared to classical stool concentration methods such as formal-ether methods. Hence, the sensitivity of the fully automated systems tends to be lower than expert manual microscopy of concentrated stool specimens [16–18]. The systems that only automatically examine pre-prepared stool slides still require the preparation of stool slides. As this is often manually performed, this is more costly, but it does allow the use of optimal concentration methods and thus a better performance. The Techcyte system was technically thoroughly examined, which demonstrated a high accuracy for a large set of well-characterized stool specimens [14]. However, the performance of the Techcyte system was not evaluated in clinical practice, nor in direct comparison with expert manual examination, which prohibits proper comparison of the clinical performance of the Techcyte and ParaScout systems.

## Limitations

Most available and validated stool specimens were prepared from formalin-preserved stool and not from concentrated fractions that are usually examined for ova and cysts (e.g. prepared by formol-ether concentration methods). In addition, some stool samples had been stored for a long time (years), which could have changed the morphology (e.g. *Strongyloides*). These factors probably affected the performance of microscopic examination negatively, and therefore, the performance of both ParaScout and manual examination might be underestimated.

For this study, the ParaScout algorithm 5.4.8 was used, as it demonstrated superior performance in pre-study comparisons conducted prior to validation. In the meantime, newer versions have been implemented with better performance after further extension of the set of images for training of the model in combination with refinement of the annotations, including the addition of protozoan species. Hence, these newer algorithm versions improve the ParaScout predictions of suspected structures, and thereby it is likely that the performance of the ParaScout system in AI-predictive mode will also be improved. For a full clinical validation, a prospective clinical cohort study is required. However, given the low prevalence of gastro-intestinal helminth infections in high-income countries, such as the Netherlands, this prospective clinical cohort study is still ongoing.

## Supplementary Material

Revised Supplementary Material 1 Development of the ParaScout AI Predictive System.docx

coi_disclosure RK.docx

Revised Supplementary Material 5 Stability analysis.docx

coi_disclosure Chris Tieken.docx

Revised Feoktistov et al Suppl data 4 full data sheet.xlsx

coi_disclosure_SS.docx

Revised Supplementary Material 7 False result analysis.docx

Revised Supplementary Material 8 Determination of final result efficiency of ParaScout.docx

coi_disclosure CMI ParaScout JJVH.docx

coi_disclosure_Parascout_RZ.docx

coi_disclosure_LvL.docx

Feoktistov et al Suppl data 3 List of helminth species.pdf

Feoktistov et al Suppl data 6 performance of ParaScout.pdf

coi_disclosure Ihor Feoktistov.docx
