# Supplementary material for: Development and validation of the AI-predictive ParaScout in-vitro diagnostic (IVD) system for the microscopic detection of gastro-intestinal helminths in stool
Source: Emerg Microbes Infect. 2026 Jul 1;15(1):2698240. doi: 10.1080/22221751.2026.2698240 (PMC13366647; doi:10.1080/22221751.2026.2698240)
Supplement: Revised Supplementary Material 1 Development of the ParaScout AI Predictive System.docx [file TEMI_A_2698240_SM7954.docx]

## **Supplementary Material 1:**

## **Development of the ParaScout AI-Predictive System**

## **1. System Overview**

The ParaScout in vitro diagnostic (IVD) system (Leven Vision B.V., Leiden, the Netherlands) is a complete end-to-end solution that combines automated microscope scanning, artificial intelligence, and intuitive user interface to standardize helminth detection in stool specimens. The system integrates a commercially available microscope scanner (Grundium Ocus 40, Tampere, Finland) with cloud-based AI interpretation and a dedicated digital review interface, addressing the two primary limitations of traditional parasitology microscopy: labor intensity and observer dependency.

The complete system workflow (Figure 1, main text) includes specimen preparation, automated scanning, AI-powered object detection, digital presentation of suspected structures through an user interface, and expert confirmation. ParaScout was developed following Good Machine Learning Practice principles within an IVDR-compliant quality framework, with integrated software development ensuring seamless laboratory workflow integration.

## **2. Training Dataset Preparation**

### **2.1 Sample Collection**

The training dataset comprised formalin-preserved stool specimens collected over a 20-year period from multiple sources: routine clinical submissions, External Quality Assessment (EQA) programs, purified helminth eggs spiked into negative stool matrices, and educational reference materials from Dutch national expertise centers (LUMC and Erasmus MC). Specimens originated from diverse geographic regions (Europe, Asia, Africa) to capture natural morphological variation across parasite populations and host demographics.

### **2.2 List of Helminth Species**

The algorithm was trained to detect 15 helminth species representing the clinically most relevant gastrointestinal helminths in both high-income and endemic settings: *Ascaris* spp., *Capillaria* spp., *Diphyllobothrium* spp., *Enterobius vermicularis*, *Fasciola* spp., hookworms (*Necator americanus* and *Ancylostoma* spp.), *Hymenolepis diminuta*, *Hymenolepis nana*, *Schistosoma haematobium*, *S. japonicum*, *S. mansoni*, *Strongyloides stercoralis*, *Taenia* spp., *Trichostrongylus* spp., and *Trichuris trichiura*.

**2.3 Image Acquisition and Annotation**

Stool specimens were prepared as wet mount preparations on microscope slides with 22×22mm coverslips and scanned using the Grundium Ocus 40 at standardized illumination and multi-focal-plane settings. The resulting dataset comprised 92,757 annotated high-resolution images.

A semi-automated annotation workflow was used: intermediate model versions generated preliminary predictions for known species, which were then reviewed and corrected. New species or challenging cases were annotated manually. This human-in-the-loop approach maintained annotation quality while speeding up dataset growth.

## **3. Neural Network Architecture**

ParaScout employs a single-shot detection architecture optimized for multi-class object detection in high-resolution microscopy images. The proprietary model incorporates:

- **Multi-scale feature extraction**: Enables detection of objects ranging from large trematode eggs (>100 µm) to small protozoa (<5 µm) within the same field of view.
- **Class-specific prediction heads**: Generate simultaneous species classification and spatial localization (bounding boxes) for each detected object.
- **Confidence scoring**: Outputs probabilistic confidence estimates enabling threshold-based quality control.

This architecture was selected for its computational efficiency and ability to process whole-slide images in clinically acceptable timeframes.

## **4. Model Training and Validation**

### **4.1 Dataset Partitioning**

The annotated dataset was partitioned into training (61.1%), validation (24.2%), and test (14.7%) subsets using stratified splitting to maintain class distribution. Critically, all images from the same physical slide were assigned to the same subset, preventing data leakage and ensuring that model evaluation reflected performance on completely unseen images.

### **4.2 Training Strategy and Model Selection**

Models were trained using standard backpropagation algorithms for deep neural network optimization with data augmentation (rotations, brightness/contrast adjustments, geometric transformations) to enhance robustness to specimen preparation variability. Model selection was based on mean Average Precision (mAP@IoU=0.50:0.95) on the validation set, with the best-performing model evaluated on the held-out test set to provide unbiased performance estimates.

Performance was systematically assessed using confusion matrices to identify species-specific misclassification patterns (e.g., *Ascaris* vs. hookworm discrimination, *Schistosoma* species differentiation). Comparative metrics across model versions guided targeted refinements, with each iteration benchmarked against predecessor performance to ensure continuous improvement without regression.

### **4.3 Performance Metrics**

Primary metrics included:

- **Mean Average Recall (mAR)**: Prioritizing clinical sensitivity
- **Species-specific F1 scores**: Balancing precision and recall for each helminth class
- **False positive rate**: Quantifying specificity and expert review burden

Optimal confidence thresholds were determined per species by maximizing F1 scores, allowing fine-grained calibration between sensitivity and specificity based on clinical importance.

**4.4 Internal Validation Protocol and Model Acceptance Criteria**

Each candidate model undergoes internal validation on a dedicated slide-level dataset, which is entirely separate from the training and validation sets. A model is accepted for clinical deployment only if it achieves 100% sensitivity at a confidence threshold of 0.6 on this dataset. Models that fail this criterion receive experimental status and are excluded from patient-facing workflows.

## **5. Iterative Refinement: Addressing Real-World Challenges**

Natural specimen rarity created a substantial imbalance, with some species represented by <500 examples. However, performance analysis demonstrated that annotation quality and image clarity were more critical than raw sample size, a finding that guided resource allocation towards quality improvement rather than simple dataset expansion.

### **5.2 Artefact Discrimination**

Early model versions exhibited over-detection of debris, pollen grains, and crystalline structures resembling helminth eggs and larvae. False positive identifications were systematically reintroduced as negative training examples in targeted retraining cycles, progressively enhancing specificity without compromising sensitivity.

### **5.3 Image Quality Management**

Image quality heterogeneity (due to preparation inconsistencies, aged specimens, or scanning artifacts) was the primary determinant of model errors. To ensure robustness, safety, and alignment with responsible AI paradigms, a multilayer quality-assurance framework was implemented:

- **Embedding-based outlier detection** to automatically surface morphologically atypical, low-fidelity, or poorly prepared specimens that could distort feature distributions.
- **Cross-validation error analysis**: to pinpoint systematically misclassified classes or sample clusters, enabling targeted expert review and early detection of hidden dataset biases.
- **Manual quality audits**: Expert parasitologists reviewed low-confidence predictions to identify systematic preparation or scanning issues.
- **Documentation and traceability protocols:** ensuring that every flagged sample, correction, and decision path was captured for auditability and model governance.

The model was trained and evaluated within a responsible AI framework that emphasized data integrity, human-in-the-loop validation, bias mitigation, and transparent quality controls. This quality-first, governance-aware approach improved model accuracy and generalizability far more effectively than naïve dataset expansion, reinforcing that rigorous data curation and expert oversight are foundational in medical AI development.

## **6. Clinical Implementation: AI-Predictive Mode**

### **6.1 Threshold Selection**

Receiver Operating Characteristic (ROC) analysis of model v5.4.8 on validation data yielded an area under the curve (AUC) of 0.997. A confidence threshold of 0.6 was selected for clinical deployment, achieving maximum sensitivity (100%, no false negatives) while maintaining a manageable false positive rate suitable for expert digital review.

### **6.2 Hybrid AI-Expert Workflow**

In AI-predictive mode, ParaScout highlights suspected structures above the confidence threshold for human expert confirmation. This hybrid approach combines the consistency of AI (complete slide coverage, no fatigue-related errors) with human expertise (morphological nuance, artefact discrimination).

## **7. Version Control and Quality Assurance**

Each algorithm iteration was assigned a version number (e.g., v5.4.8 used in this study). New versions underwent rigorous internal validation on quality control specimens before deployment. Performance metrics were continuously monitored across versions to prevent regression.

## **8. Ongoing Development**

After each validation cycle, misclassified structures were re-evaluated by expert parasitologists, re-annotated if necessary, and fed back into the next training round. This human-in-the-loop feedback loop ensured constant improvement and adaptation to real-world sample variability.

The validated models are continuously updated and integrated into the ParaScout IVD platform as a traceable and deployable software component. Each model version operates within a controlled release process with documented version management, software verification and IVDR-compliant traceability, ensuring that the platform remains stable while the underlying AI models evolve.

**Supplementary Material 2:** [**Movie demonstrating the use of ParaScout**](https://eur.cloud.panopto.eu/Panopto/Pages/Viewer.aspx?id=3222bc08-e236-429a-96f6-b3c00095e3f9)**.**

https://eur.cloud.panopto.eu/Panopto/Pages/Viewer.aspx?id=3222bc08-e236-429a-96f6-b3c00095e3f9
