# Supplementary material for: Development and validation of the AI-predictive ParaScout in-vitro diagnostic (IVD) system for the microscopic detection of gastro-intestinal helminths in stool
Source: Emerg Microbes Infect. 2026 Jul 1;15(1):2698240. doi: 10.1080/22221751.2026.2698240 (PMC13366647; doi:10.1080/22221751.2026.2698240)
Supplement: Revised Supplementary Material 5 Stability analysis.docx [file TEMI_A_2698240_SM7952.docx]

# Supplementary Material 5: Analysis of stability before and after manual examination

| **Slide #** | **Initial predictions** | **N initial** | **Post-examination predictions** | **N post** | **Δ** | **Stability** |
| --- | --- | --- | --- | --- | --- | --- |
| 3 | General - 72  Ascaris lumbricoides - 38  Hookworm - 4  Hymenolepis nana - 30 | 72 | General - 98  Ascaris lumbricoides - 55  Hookworm - 6  Hymenolepis nana - 37 | 98 | +26 | 136% |
| 4 | General - 18  Ascaris lumbricoides - 18 | 18 | General - 27  Ascaris lumbricoides - 27 | 27 | +9 | 150% |
| 6 | General - 0 | 0 | General - 0 | 0 | 0 | 7 |
| 2 | General - 0 | 0 | General - 0 | 0 | 0 | n.r. |
| 35 | General - 57  Ascaris lumbricoides - 57 | 57 | General - 79  Ascaris lumbricoides - 79 | 79 | +22 | 139% |
| 38 | General - 262  Ascaris lumbricoides - 182  Trichuris trichiura - 80 | 262 | General - 277  Ascaris lumbricoides - 192  Trichuris trichiura - 85 | 277 | +15 | 106% |
| 37 | General - 1  Hymenolepis diminuta - 1 | 1 | General - 1  Hymenolepis diminuta - 1 | 1 | 0 | 100% |
| 45 | General - 17  Strongyloides stercoralis - 17 | 17 | General - 32  Strongyloides stercoralis - 32 | 32 | +15 | 188% |
| 47 | General - 105  Ascaris lumbricoides - 105 | 105 | General - 120  Ascaris lumbricoides - 120 | 120 | +15 | 114% |
| 55 | General - 0 | 0 | General - 0 | 0 | 0 | n.r. |
| 58 | General - 81  Fasciola spp. - 81 | 81 | General - 96  Fasciola spp. - 96 | 96 | +15 | 119% |
| 60 | General - 2  Enterobius vermicularis - 2 | 2 | General - 3  Enterobius vermicularis - 3 | 3 | +1 | 150% |
| 62 | General - 9  Schistosoma mansoni - 9 | 9 | General - 8  Schistosoma mansoni - 8 | 8 | -1 | 89% |
| 63 | General - 1  Schistosoma japonicum - 1 | 1 | General - 1  Schistosoma japonicum - 1 | 1 | 0 | 100% |
| 68 | General - 14  Hookworm - 1  Schistosoma mansoni - 13 | 14 | General - 13  Hookworm - 1  Schistosoma mansoni - 12 | 13 | -1 | 93% |
| 70 | General - 131  Hookworm - 111  Hymenolepis diminuta - 20 | 131 | General - 132  Hookworm - 111  Hymenolepis diminuta - 21 | 132 | +1 | 101% |
| 112 | General - 0 | 0 | General - 0 | 0 | 0 | n.r. |
| 131 | General - 34  Ascaris lumbricoides - 20  Hookworm - 1  Hymenolepis nana - 13 | 34 | General - 90  Ascaris lumbricoides - 45  Hookworm - 3  Hymenolepis nana - 42 | 90 | +56 | 265% |
| 133 | General - 199  Diphyllobothrium spp. - 199 | 199 | General - 204  Diphyllobothrium spp. - 204 | 204 | +5 | 103% |
| 144 | General - 22  Hookworm - 22 | 22 | General - 22  Hookworm - 22 | 22 | 0 | 100% |
| 164 | General - 50  Fasciola spp. - 50 | 50 | General - 47  Fasciola spp. - 47 | 47 | +3 | 94% |
| 165 | General - 0 | 0 | General - 0 | 0 | 0 | n.r. |
| 168 | General - 15  Schistosoma mansoni - 15 | 15 | General - 15  Schistosoma mansoni - 15 | 15 | 0 | 100% |
| 192 | General - 10  Hymenolepis nana - 10 | 10 | General - 10  Hymenolepis nana - 10 | 10 | 0 | 100% |
| 193 | General - 1  Hookworm - 1 | 1 | General - 1  Hookworm - 1 | 1 | 0 | 100% |
| 194 | General - 19  Schistosoma mansoni - 13  Strongyloides stercoralis - 6 | 19 | General - 19  Schistosoma mansoni - 12  Strongyloides stercoralis - 7 | 19 | 0 | 100% |
| 294 | General - 15  Schistosoma mansoni - 8  Strongyloides stercoralis - 7 | 15 | General - 15  Schistosoma mansoni - 7  Strongyloides stercoralis - 8 | 15 | 0 | 100% |
| 341 | General - 0 | 0 | General - 0 | 0 | 0 | n.r. |
| 362 | General - 13  Enterobius vermicularis - 13 | 13 | General - 13  Enterobius vermicularis - 13 | 13 | 0 | 100% |
| 371 | General - 2  Hymenolepis diminuta - 2 | 2 | General - 2  Hymenolepis diminuta - 2 | 2 | 0 | 100% |
| 377 | General - 1  Hymenolepis diminuta - 1 | 1 | General - 3  Hymenolepis diminuta - 3 | 3 | +2 | 300% |
| 693 | General - 135  Ascaris lumbricoides - 111  Capillaria spp. - 4  Schistosoma mansoni - 20 | 135 | General - 122  Ascaris lumbricoides - 98  Capillaria spp. - 6  Schistosoma mansoni - 18 | 122 | +13 | 90% |
| 752 | General - 18  Enterobius vermicularis - 18 | 18 | General - 23  Enterobius vermicularis - 23 | 23 | +5 | 128% |
| 776 | General - 2  Fasciola spp. - 2 | 2 | General - 2  Fasciola spp. - 2 | 2 | 0 | 100% |
| 777 | General - 0 | 0 | General - 0 | 0 | 0 | n.r. |
| 778 | General - 43  Hookworm - 36  Hymenolepis diminuta - 7 | 43 | General - 44  Hookworm - 37  Hymenolepis diminuta - 7 | 44 | +1 | 102% |
| 810 | General - 20  Hookworm - 17  Trichuris trichiura - 3 | 20 | General - 19  Hookworm - 16  Trichuris trichiura - 3 | 19 | -1 | 95% |
| 892 | General - 0 | 0 | General - 0 | 0 | 0 | n.r. |
| 894 | General - 9  Schistosoma mansoni - 9 | 9 | General - 9  Schistosoma mansoni - 9 | 9 | 0 | 100% |
| 899 | General - 3  Fasciola spp. - 3 | 3 | General - 4  Fasciola spp. - 4 | 4 | +1 | 133% |
| 900 | General - 2  Fasciola spp. - 2 | 2 | General - 2  Fasciola spp. - 2 | 2 | 0 | 100% |
| 910 | General - 34  Hookworm - 34 | 34 | General - 30  Hookworm - 30 | 30 | -4 | 88% |
| 945 | General - 14  Schistosoma mansoni - 14 | 14 | General - 14  Schistosoma mansoni - 14 | 14 | 0 | 100% |
| 947 | General - 21  Ascaris lumbricoides - 21 | 21 | General - 19  Ascaris lumbricoides - 19 | 19 | -2 | 90% |
| 950 | General - 3  Schistosoma japonicum - 3 | 3 | General - 6  Schistosoma japonicum - 6 | 6 | +3 | 200% |
| 952 | General - 0 | 0 | General - 0 | 0 | 0 | n.r. |
| 955 | General - 0 | 0 | General - 0 | 0 | 0 | n.r. |
| 956 | General - 13  Strongyloides stercoralis - 13 | 13 | General - 16  Strongyloides stercoralis - 16 | 16 | +3 | 123% |
| 958 | General - 1  Schistosoma japonicum - 1 | 1 | General - 3  Schistosoma japonicum - 3 | 3 | +2 | 300% |
| 993 | General - 8  Hookworm - 8 | 8 | General - 8  Hookworm - 8 | 8 | 0 | 100% |

## S2. Cases with a stability higher than 120% or less than 80%.

Detailed analysis was performed for 12 specimens for which the stability score was higher than 120%. In all other cases the stability scored was between 88% and 119%, and can be considered under normal confidence noise. There are also no cases with stability less than 80%, which shows that slide quality generally increased in the scans after manual examination compared to before manual examination. The table below shows a summary of the results of the performed discrepancy analysis, after which details are provided.

| **Slide ID** | **N initial** | **N post** | **Stability (%)** | **Δ** | **Category** | **Primary cause** |
| --- | --- | --- | --- | --- | --- | --- |
| 3 | 72 | 98 | 136% | +26 | A | Settling - improved object definition |
| 4 | 18 | 27 | 150% | +9 | A | Settling - improved object definition |
| 35 | 57 | 79 | 139% | +22 | A | Settling - improved object definition |
| 45 | 17 | 32 | 188% | +15 | A | Settling - objects not previously visible |
| 60 | 2 | 3 | 150% | +1 | B | Reorientation - object flattened between scans |
| 131 | 34 | 90 | 265% | +56 | A | Settling, with minor loss in initially detected set |
| 377 | 1 | 3 | 300% | +2 | A | Settling - objects not previously visible |
| 752 | 18 | 23 | 128% | +5 | A | Settling - reduced obstruction by debris |
| 899 | 3 | 4 | 133% | +1 | C | Scanning stitching artifact on first scan reducing confidence |
| 950 | 3 | 6 | 200% | +3 | D | Low confidence due to settling process |
| 956 | 13 | 16 | 123% | +3 | A | Settling - improved object definition |
| 958 | 1 | 3 | 300% | +2 | D | Reclassification - objects misclassified on first scan |

## S3. Grouped analysis of causes

### S3.1 Category A: Sedimentation of slide media between scans

The majority of the observed stability increases (slides 3, 4, 35, 45, 131, 377, 752, and 956) are most likely due to settling down of the suspended particles in the wet-mounted media between the initial and post-examination scans. Since specimens were prepared just before examination the objects in the specimens will sedimentate over time in the slide and some of them were out of focus in initial scans while they had settled to the focus plane at the time of the post-examination scans.

Representative cases:

**Slide 45 (188%) - *Strongyloides stercoralis*.**

Several *Strongyloides stercoralis* larvae were not visible at all on the initial scan and became distinctly visible in the scan after manual examination.

| Initial  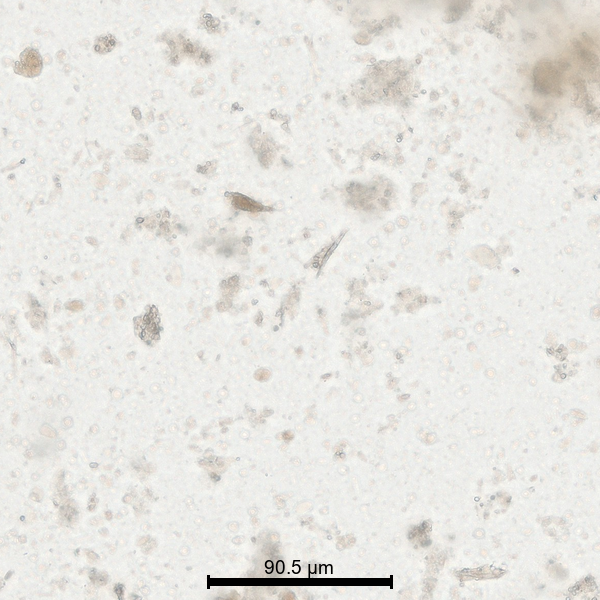  Not detected by ParaScout | Post-examination  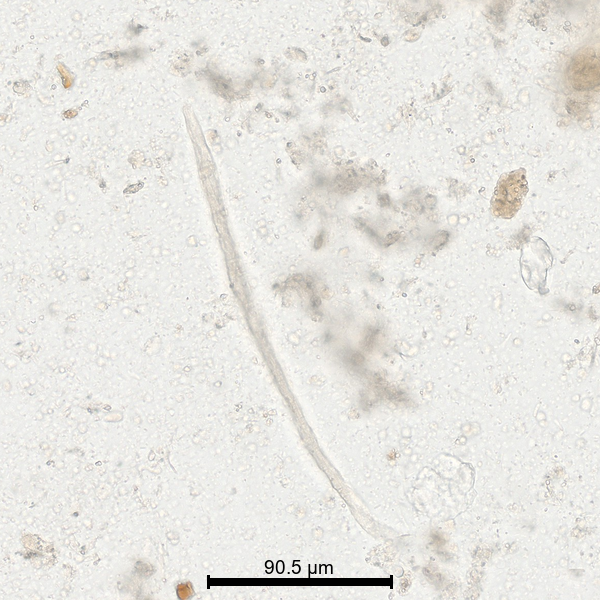  Detected by ParaScout |
| --- | --- |
| 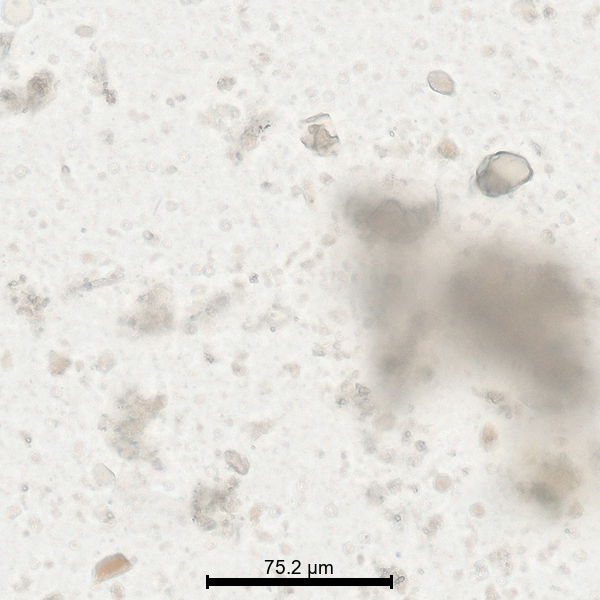  Not detected by ParaScout | 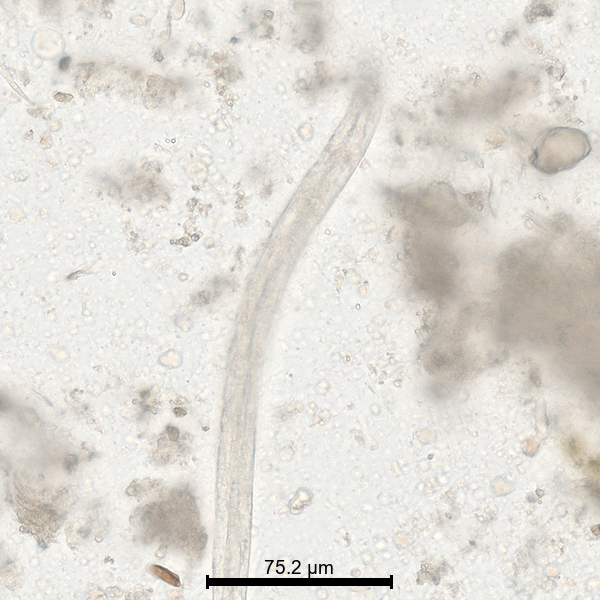  Detected by ParaScout |

**Slide 3 (136%)** - ***Ascaris lumbricoides*, Hookworm, *Hymenolepos nana* and slide 4 (150%)** - ***Ascaris lumbricoides*.**

In both slides, every object detected on the initial scan was also detected on the post-examination scan. The additional detections became more in focus in the second scan after examination as a result of sedimentation to the proper focal plane.

| Initial  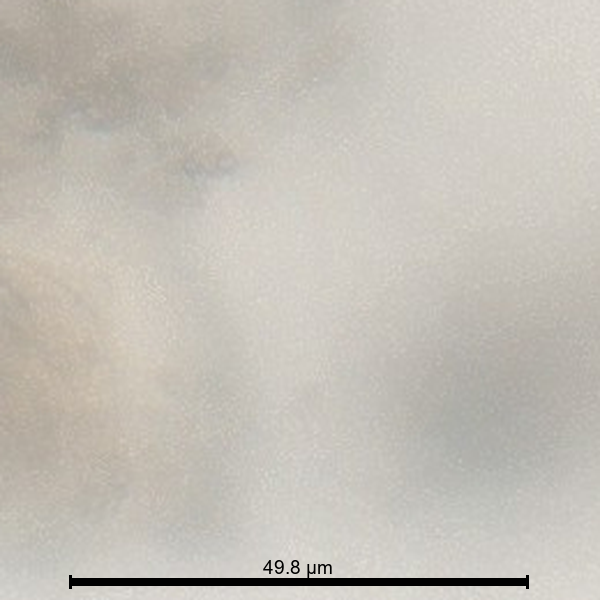  Not detected by ParaScout | Post-examination  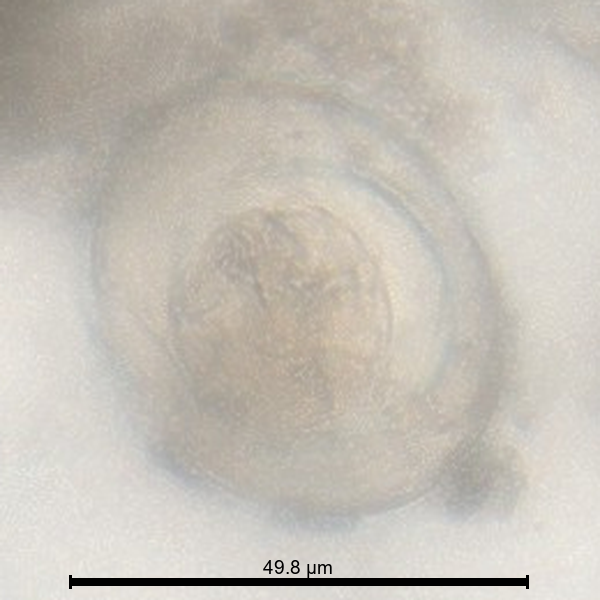  Detected by ParaScout |
| --- | --- |
| 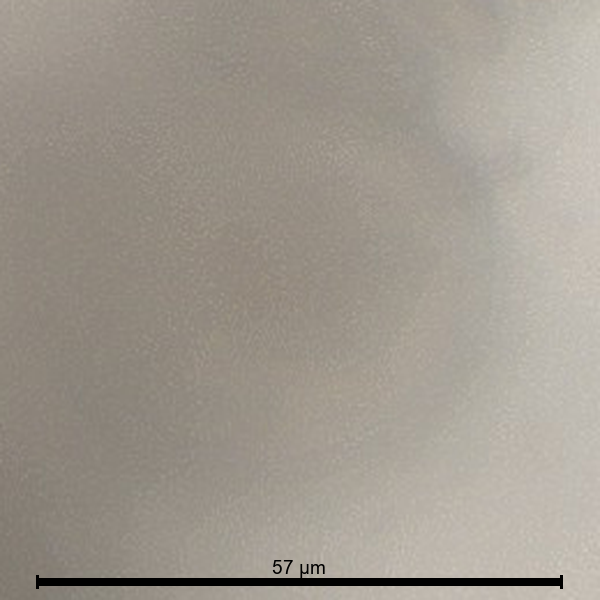  Not detected by ParaScout | 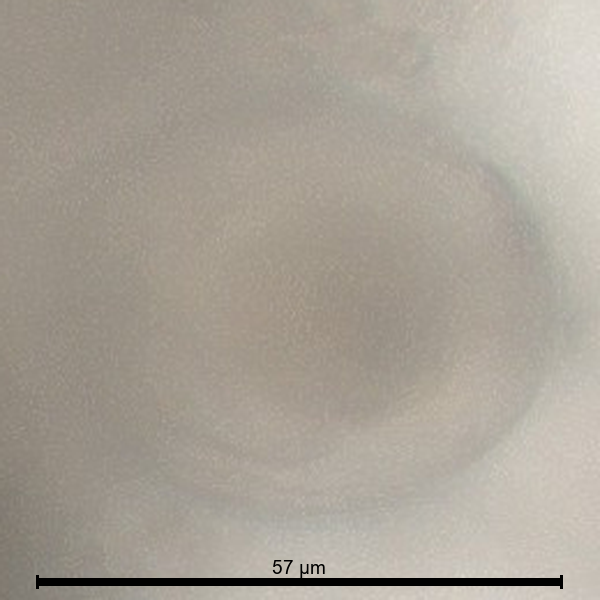  Detected by ParaScout |

**Slide 752 (128%) - *Enterobius vermicularis*.**

Five additional *Enterobius vermicuralis* eggs, which were obstructed by floating debris or were generally out of focus in the first scan, became better visible in the second scan after manual examination.

| Initial  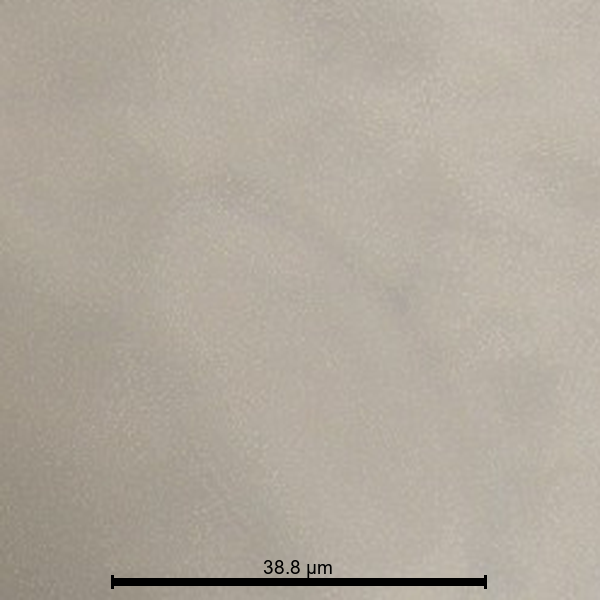  Not detected by ParaScout | Post-examination  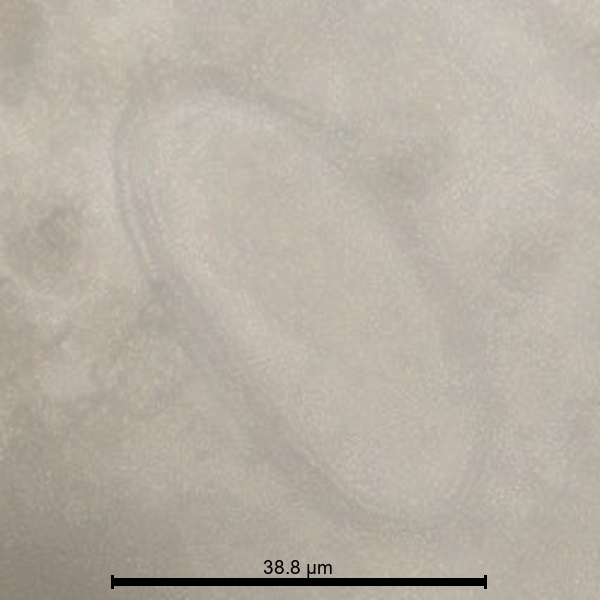  Detected by ParaScout |
| --- | --- |

**Slide 131 (265%)** - ***Ascaris lumbricoides*, Hookworm, *Hymenolepos nana*.**

This slide is the only one in Category A (Settling - improved object definition), in which a small number of Ascaris eggs detected on the initial scan were no longer detected in the second scan after examination. However, due to the overall high object count (34 → 90) this is to be expected, and the end result was better as more objects became well defined.

| Initial  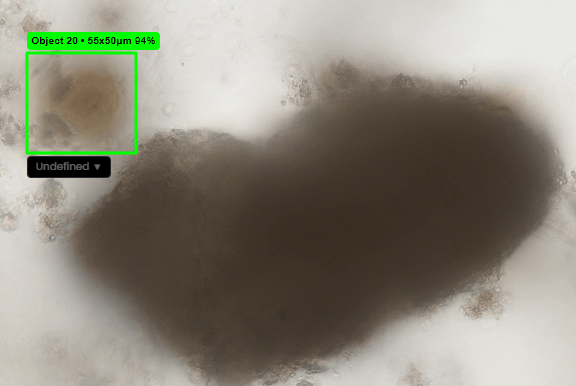  Detected by ParaScout | Post-examination  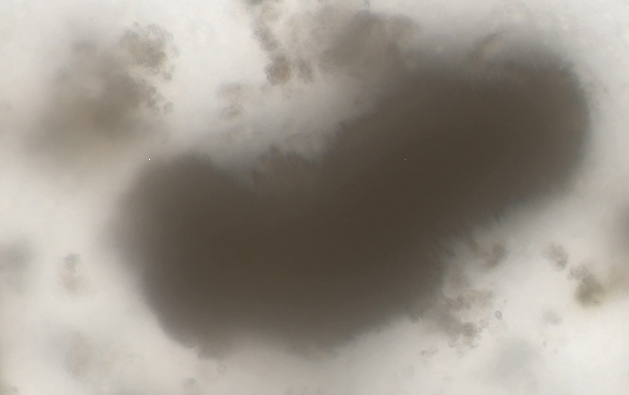  Not detected by ParaScout |
| --- | --- |

### S3.2 Category B: Reorientation of objects between scans

**Slide 60 (150%) - *Enterobius vermicularis*.**

This case can probably also be attributed to settling of *Enterobius vermicuralis* eggs between scanning before and after manual examination, but it is a bit different in the sense that the initial object is visible in the initial scan, but due to it being rotated only parts of it are in focus on each scanned layer. In the post-examination scan it lays flat, and therefore the full object is in focus in a single layer, which allows for detection.

| Initial 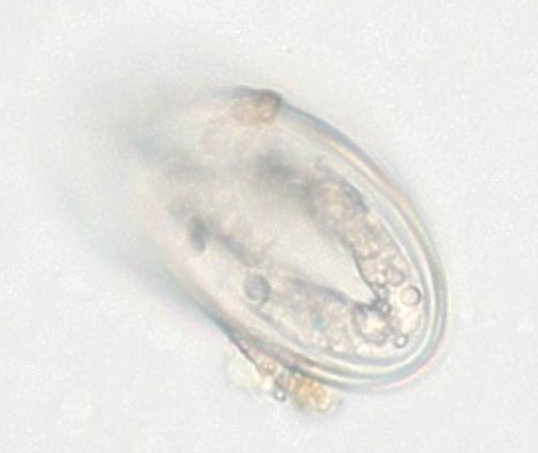  Not detected by ParaScout | Post-examination  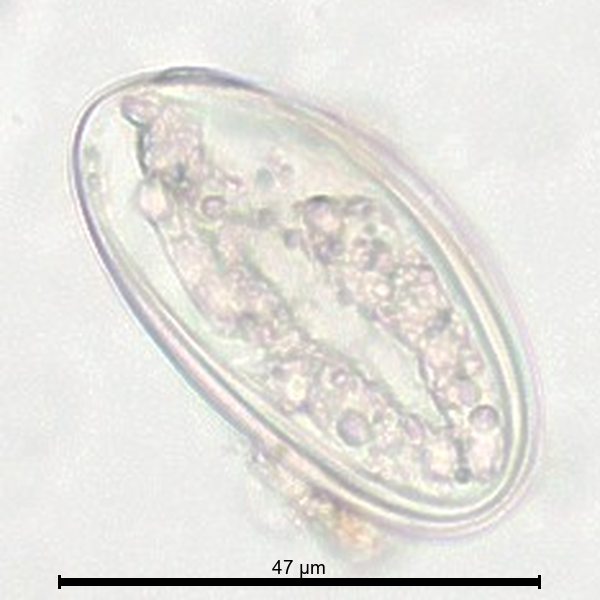  Detected by ParaScout |
| --- | --- |

### S3.3 Category C: Scanner stitching artifacts on the initial scan

**Slide 899 (133%) - *Fasciola* spp.**

This slide differs from the preceding categories in that the difference is probably attributable to an imaging artifact. The additional *Fasciola* egg was captured in between fields of view (FOVs), and moved between pictures collected during the scanning process, so the image-stitching algorithm defected the object. The model still recognised this *Fasciola* egg, but with a reduced confidence score of only 36%, which is under the confidence threshold. After the manual examination, the position of the *Fasciola* egg shifted slightly, increasing its visibility.

| Initial  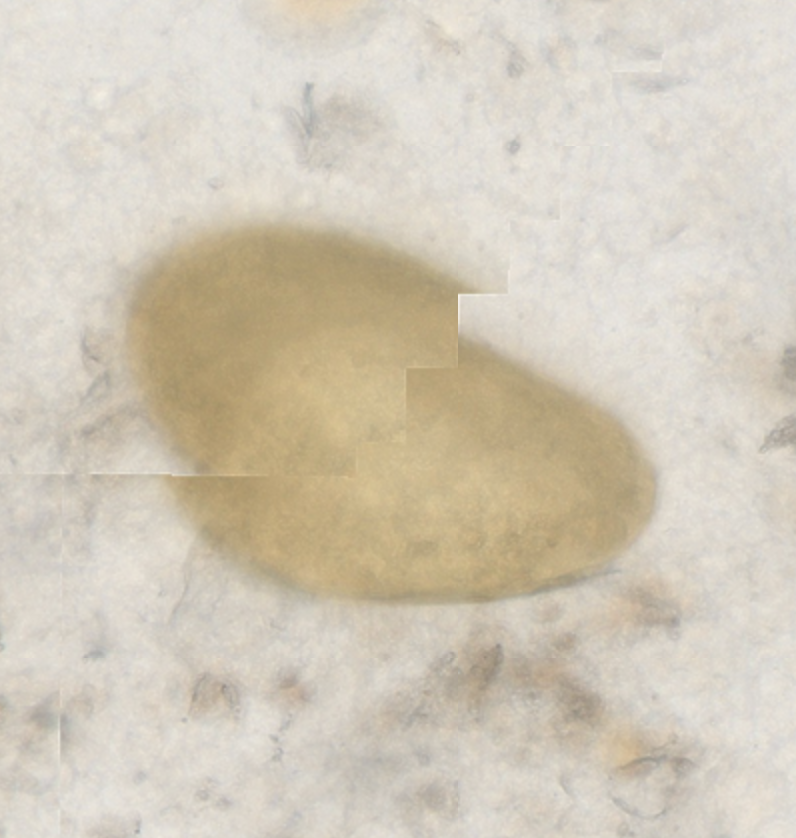  Detected by ParaScout with low confidence | Post-examination  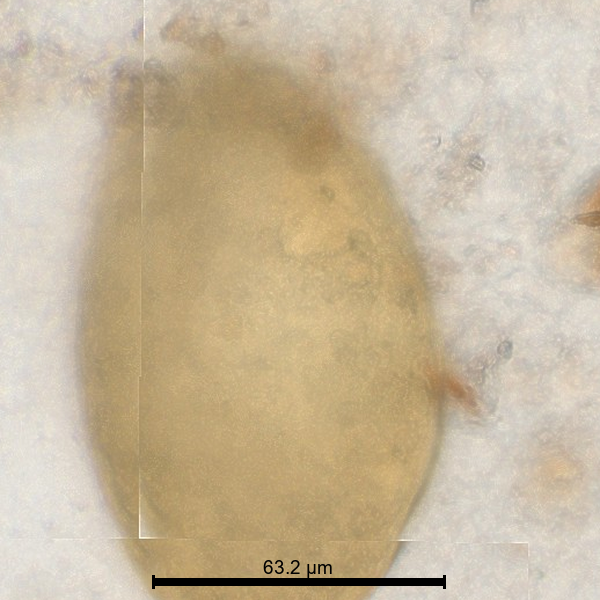  Detected by ParaScout with up confidence |
| --- | --- |

### S3.4 Category D: Confidence-threshold and classification recovery

In several instances the objects were present and detected in both scans. However due to a marginal shift in their position the detection confidence increased slightly, allowing them to pass the 60% confidence threshold for detection.

**Slide 950 (200%) - *Schistosoma japonicum*.**

Of the three additional *Schistosoma japonicum* eggs detected on the post-examination scan, one had been detected initially with a confidence of 54%, just below threshold, and the other two had been detected but misclassified as different species. After the clarity improvement between scans all three crossed the threshold and were correctly classified.

| Initial  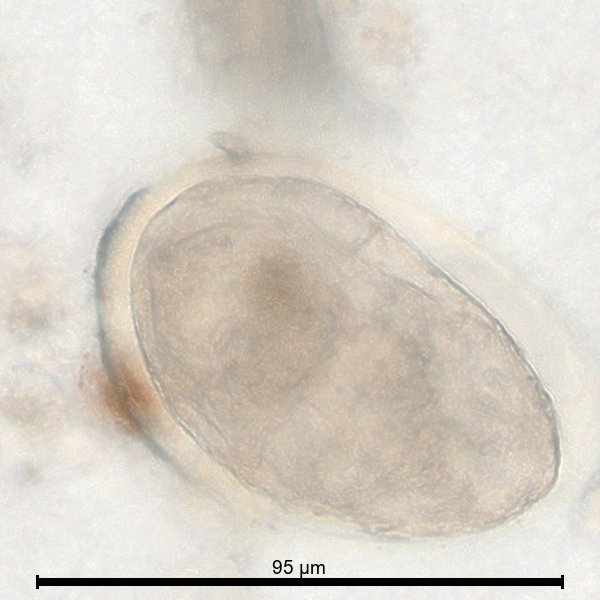  Detected as wrong class by ParaScout | Post-examination  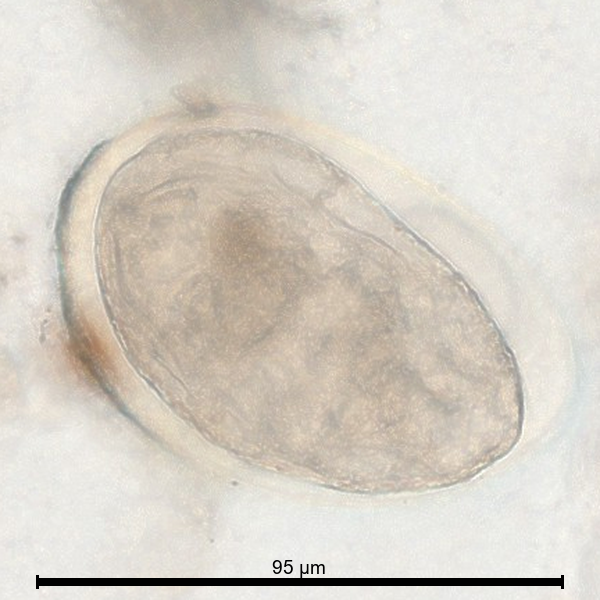  Detected by ParaScout |
| --- | --- |

**Slide 958 (300%) - *Schistosoma japonicum*.**

All three *Schisostoma japonicum* eggs identified on the post-examination scan had also been detected on the initial scan, but only one was correctly classified there; the remaining two had been assigned to other species. Once again on the second scan the visibility increased marginally allowing for correct classification.

| Initial  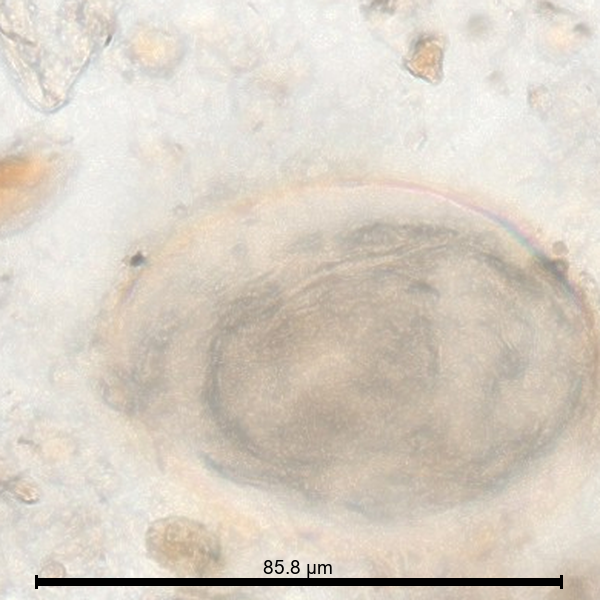  Detected as wrong class by ParaScout | Post-examination  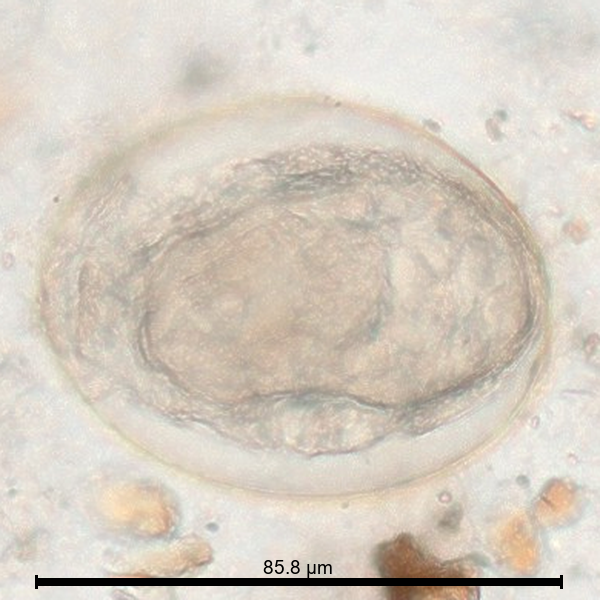  Detected by ParaScout |
| --- | --- |

## S4. Implications for slide preparation and scanning protocol

Three main conclusions can be drawn from this analysis. First, the changes between the initial and post-examination scans show consistent accuracy increase. The number of detected helminths increased while no increase in false positives was observed.

Second, the prevailing cause of stability changes can be attributed to sedimentation of suspended particle matter.

It should also be noted that the majority of the additional objects identified on the scans after manual examination were not missed by the model in an algorithmic sense: they were either not visible in the image, partially out of focus, or obscured by debris. Therefore the resulting detection confidence fell below the inclusion threshold. The model's behaviour in these cases reflects the quality of the used slide images, not instability of the model itself.
