# Supplementary material for: Development and validation of the AI-predictive ParaScout in-vitro diagnostic (IVD) system for the microscopic detection of gastro-intestinal helminths in stool
Source: Emerg Microbes Infect. 2026 Jul 1;15(1):2698240. doi: 10.1080/22221751.2026.2698240 (PMC13366647; doi:10.1080/22221751.2026.2698240)
Supplement: Revised Supplementary Material 7 False result analysis.docx [file TEMI_A_2698240_SM7948.docx]

## Supplementary data 7: False results analysis

Using a confidence threshold of 0.6, the neural network model (version 5.4.8) provided results with 100% sensitivity and 91.7% specificity. Overview of results is shown in the table below.

|  | **ParaScout detections with confidence threshold: 0.6** | | | |
| --- | --- | --- | --- | --- |
|  |  |  |  |  |
|  | **TP** | **FP** | **TN** | **FN** |
| Ascaris lumbricoides | 8 | 4 | 38 |  |
| Capillaria spp. | 1 |  | 49 |  |
| Diphyllobothrium spp. | 1 | 4 | 45 |  |
| Enterobius vermicularis | 3 | 1 | 46 |  |
| Fasciola spp. | 5 | 2 | 43 |  |
| Hookworm | 10 | 5 | 35 |  |
| Hymenolepis diminuta | 5 | 3 | 42 |  |
| Hymenolepis nana | 3 | 3 | 44 |  |
| Schistosoma haematobium |  | 3 | 47 |  |
| Schistosoma japonicum | 3 |  | 47 |  |
| Schistosoma mansoni | 8 | 6 | 36 |  |
| Strongyloides stercoralis | 4 | 20 | 26 |  |
| Taenia spp. | 10 |  | 40 |  |
| Trichostrongylus spp. |  | 5 | 45 |  |
| Trichuris trichiura | 2 | 1 | 47 |  |
|  |  |  |  |  |
| **Total** | **63** | **57** | **630** | **0** |

In order to improve the specificity of the outcome, 4 expert technicians examined the digital images of all selected structures (grouped in abnormality classes). Expert technicians reviewed objects for each abnormality group whether the presence of a certain helminth species could be confirmed, or rejected as artifacts or that manual microscopic examination would be needed to draw a final conclusion.

In most cases the selected structures contained sufficient morphological characteristics to allow helminth species identification. The figure below shows representative examples of selected structures by the ParaScout dataset.

| 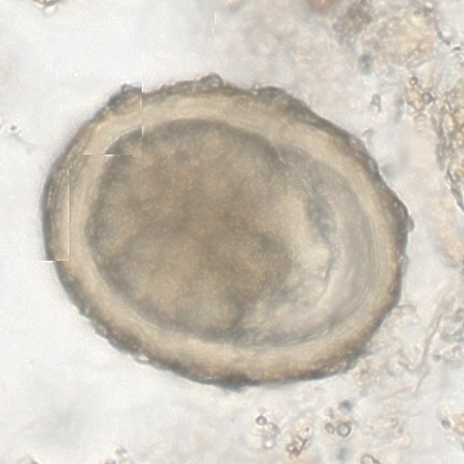 | 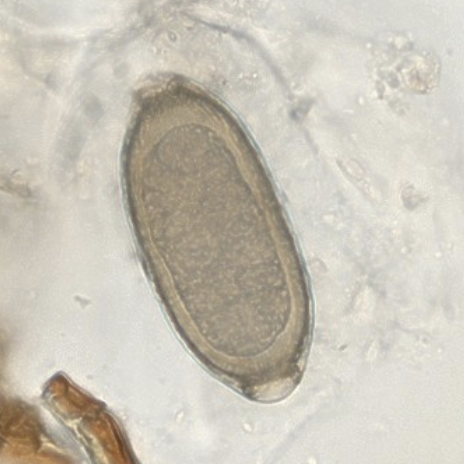 | 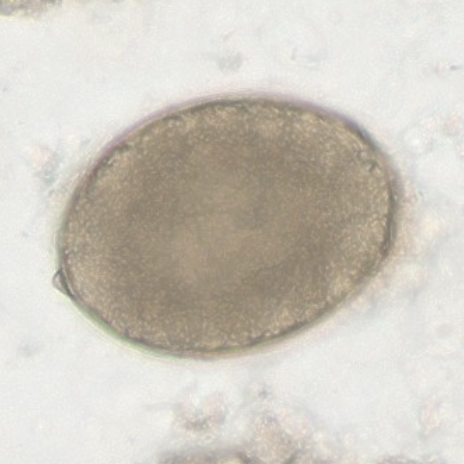 |
| --- | --- | --- |
| 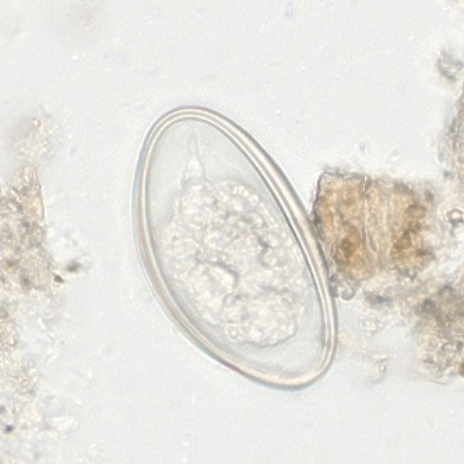 | 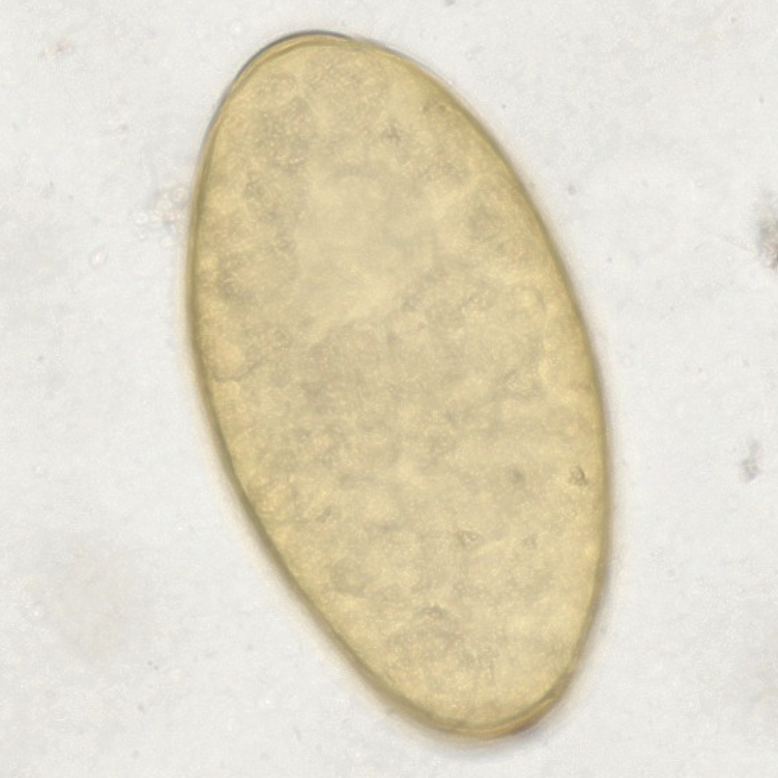 | 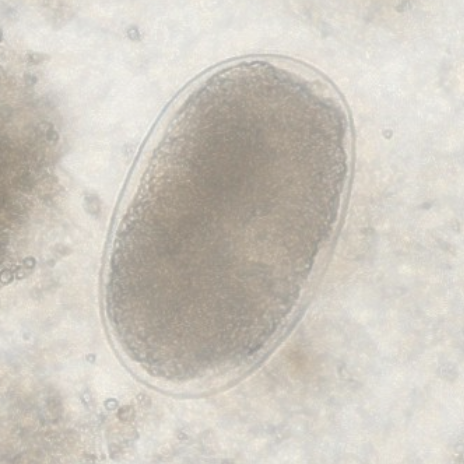 |
| 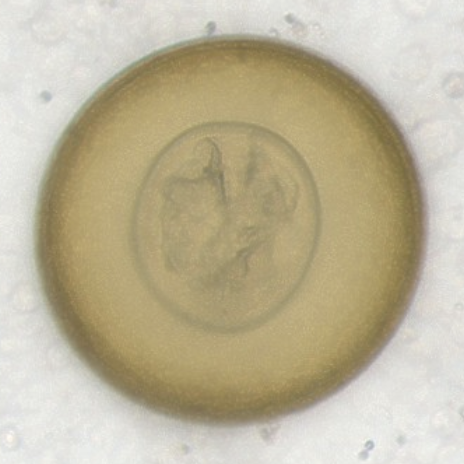 | 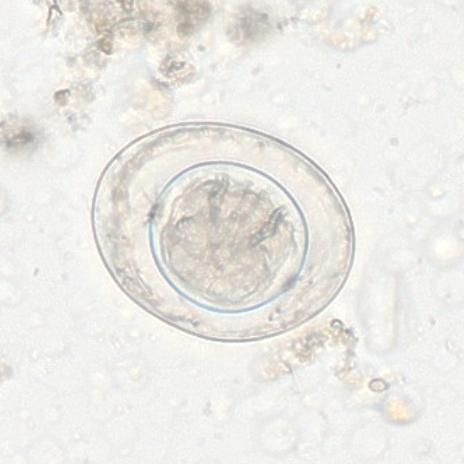 | 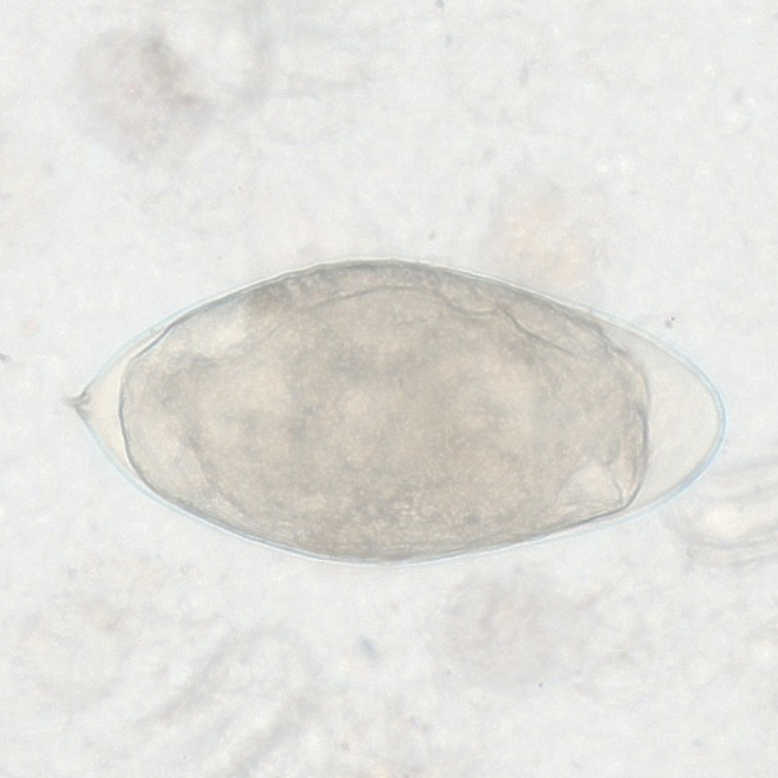 |
| 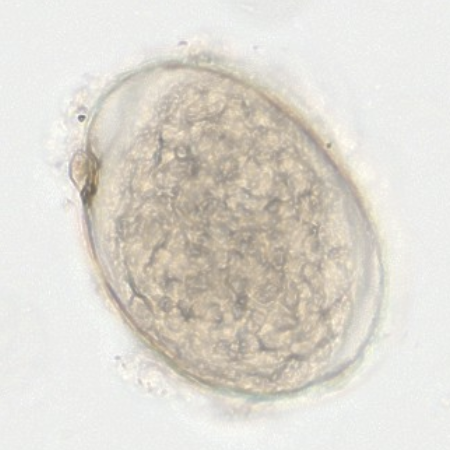 | 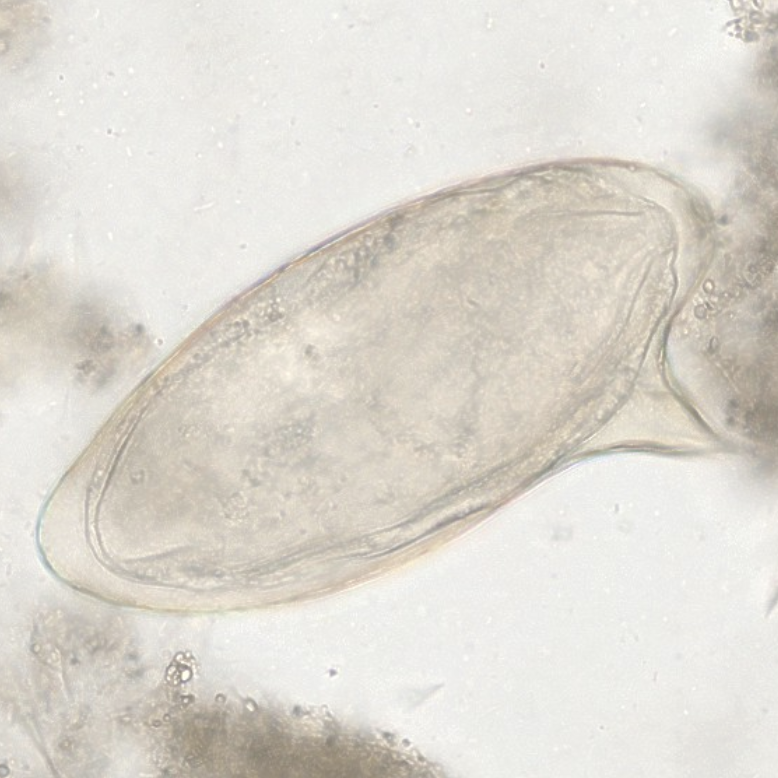 | 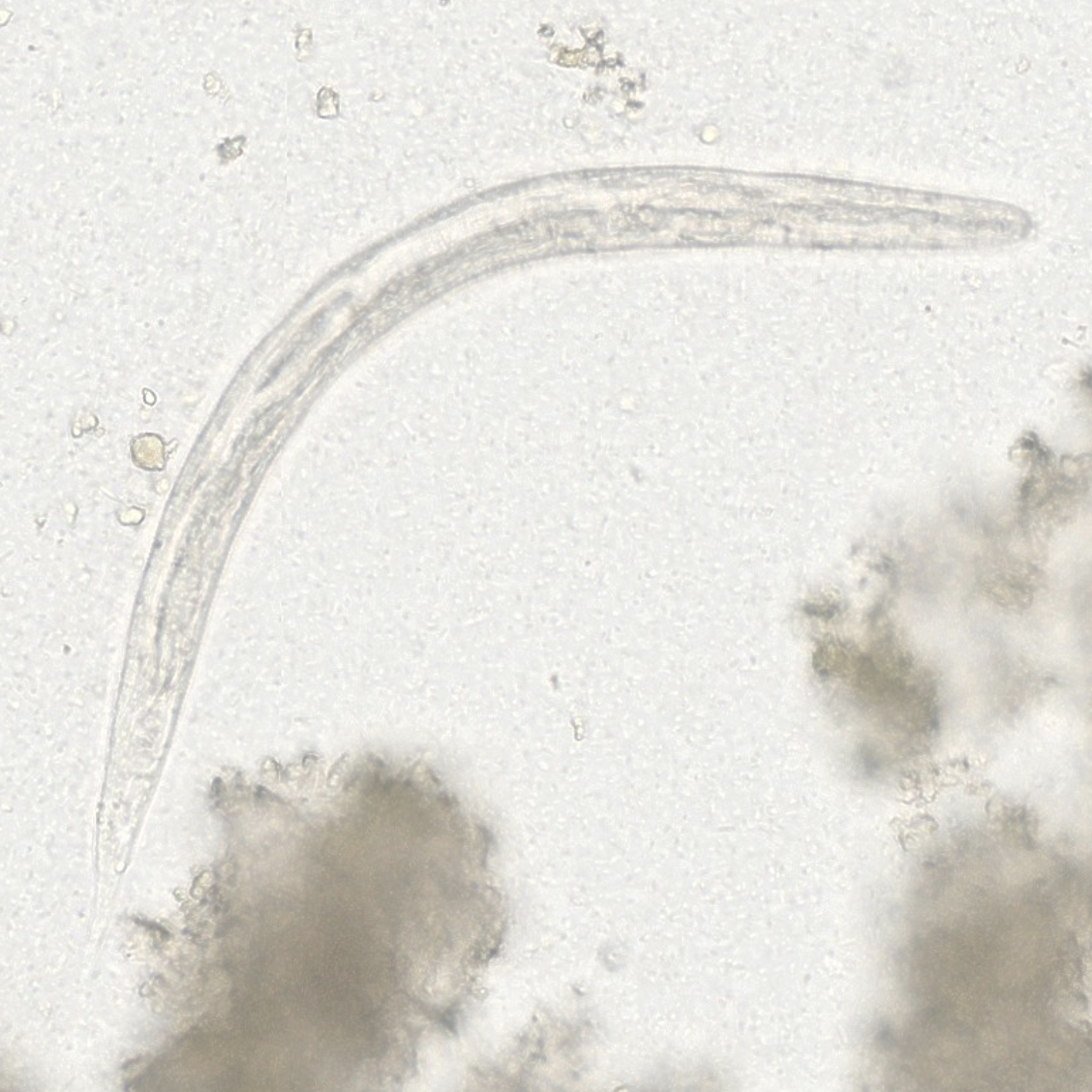 |
| 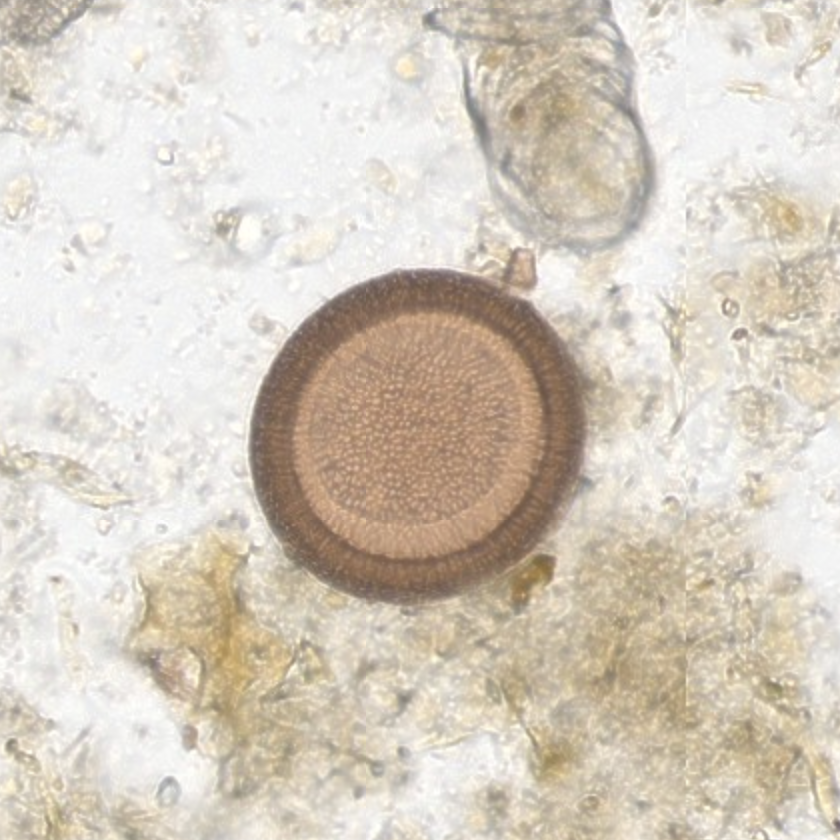 | 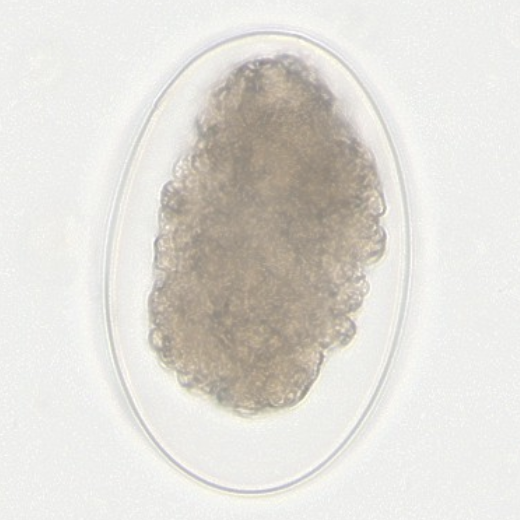 | 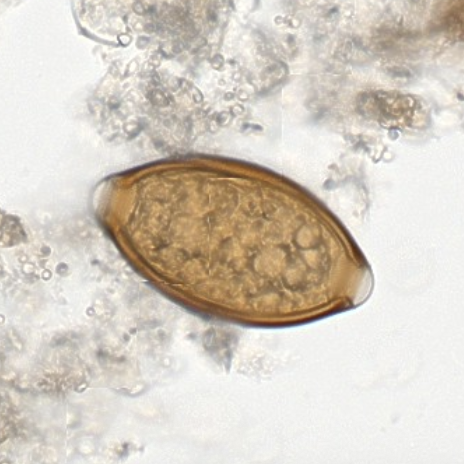 |

The results of the ParaScout AI-assisted examination with confirmation by expert technician, are shown in the table below.

|  | **AI-predictive ParaScout** | | | | | | | | | | | | | | | |
| --- | --- | --- | --- | --- | --- | --- | --- | --- | --- | --- | --- | --- | --- | --- | --- | --- |
|  | **ParaScout with Technician nr: 5** | | | | **ParaScout with Technician nr: 6** | | | | **ParaScout with Technician nr: 7** | | | | **ParaScout with Technician nr: 8** | | | |
|  | **TP** | **FP** | **TN** | **FN** | **TP** | **FP** | **TN** | **FN** | **TP** | **FP** | **TN** | **FN** | **TP** | **FP** | **TN** | **FN** |
| Ascaris lumbricoides | 8 |  | 42 |  | 8 |  | 42 |  | 8 |  | 42 |  | 8 |  | 42 |  |
| Capillaria spp. | 1 |  | 49 |  | 1 |  | 49 |  | 1 |  | 49 |  | 1 |  | 49 |  |
| Diphyllobothrium spp. | 1 |  | 49 |  | 1 | 1 | 48 |  | 1 |  | 49 |  | 1 |  | 49 |  |
| Enterobius vermicularis | 3 |  | 47 |  | 3 |  | 47 |  | 3 |  | 47 |  | 3 |  | 47 |  |
| Fasciola spp. | 5 |  | 45 |  | 5 |  | 45 |  | 5 |  | 45 |  | 5 |  | 45 |  |
| Hookworm | 10 | 1 | 39 |  | 10 |  | 40 |  | 10 |  | 40 |  | 10 |  | 40 |  |
| Hymenolepis diminuta | 4 |  | 45 | 1 | 4 |  | 45 | 1 | 5 |  | 45 |  | 4 |  | 45 | 1 |
| Hymenolepis nana | 3 |  | 47 |  | 3 |  | 47 |  | 3 |  | 47 |  | 3 |  | 47 |  |
| Schistosoma haematobium |  |  | 50 |  |  |  | 50 |  |  |  | 50 |  |  |  | 50 |  |
| Schistosoma japonicum | 3 |  | 47 |  | 3 | 1 | 46 |  | 3 |  | 47 |  | 3 |  | 47 |  |
| Schistosoma mansoni | 8 |  | 42 |  | 8 |  | 42 |  | 8 |  | 42 |  | 8 |  | 42 |  |
| Strongyloides stercoralis | 4 |  | 46 |  | 4 |  | 46 |  | 4 |  | 46 |  | 4 |  | 46 |  |
| Taenia spp. | 10 |  | 40 |  | 10 |  | 40 |  | 10 | 1 | 39 |  | 10 |  | 40 |  |
| Trichostrongylus spp. |  |  | 50 |  |  |  | 50 |  |  |  | 50 |  |  |  | 50 |  |
| Trichuris trichiura | 2 |  | 48 |  | 2 |  | 48 |  | 2 |  | 48 |  | 2 |  | 48 |  |
|  |  |  |  |  |  |  |  |  |  |  |  |  |  |  |  |  |
| **Total** | **62** | **1** | **686** | **1** | **62** | **2** | **685** | **1** | **63** | **1** | **686** | **0** | **62** | **0** | **687** | **1** |

As shown above, 4 false positive results remained and 3 false negative results appeared. These 7 false results were present in 5 slides and all 5 false results are discussed one by one below.

### Hookworm false positive. Slide #47 (1 false positive)

The intended results of slide #47 are *Ascaris lumbricoides* and *Taenia* spp.

Group #1. Objects marked as *Ascaris lumbricoides* by 4 technicians:

| 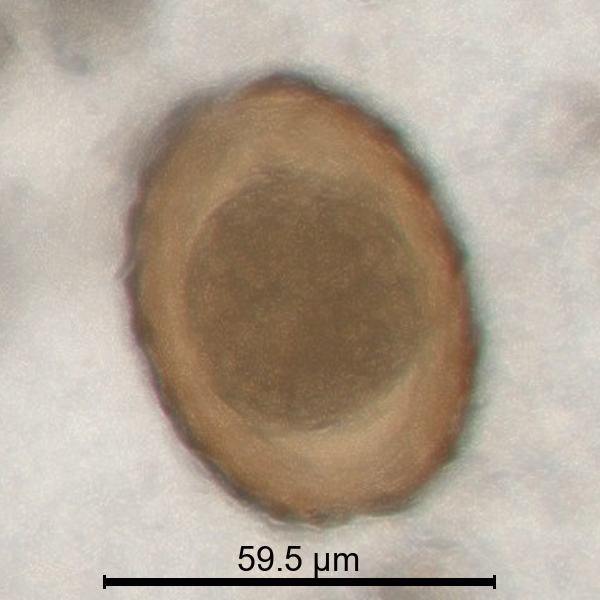 | 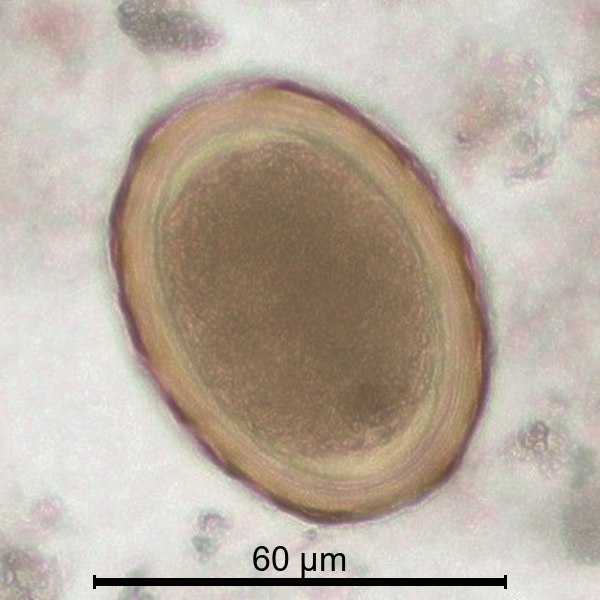 | 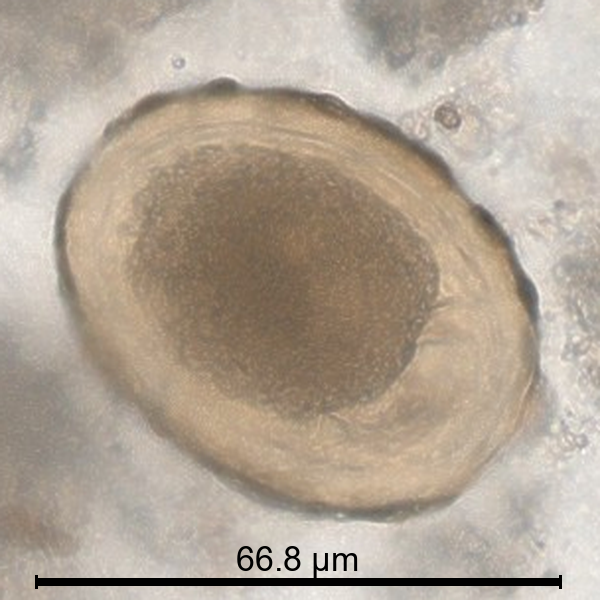 | 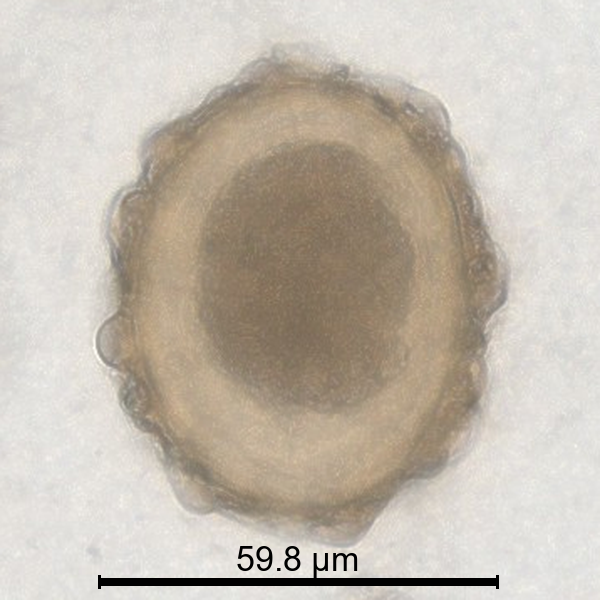 |
| --- | --- | --- | --- |

Group #2. Objects marked as *Taenia* spp. by 4 technicians:

| 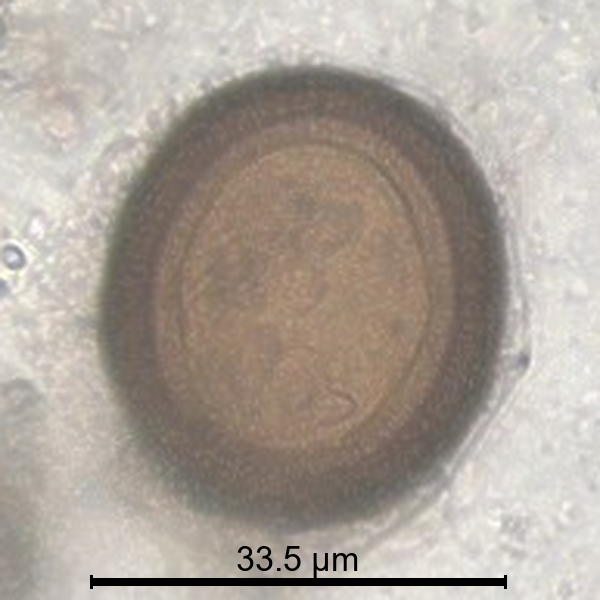 | 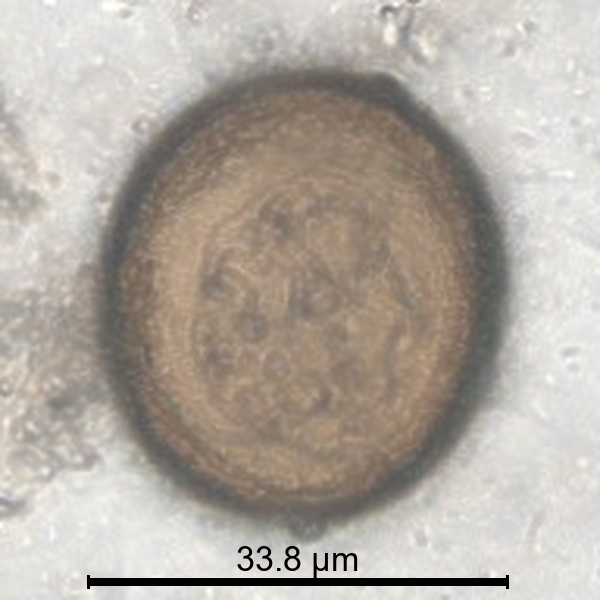 | 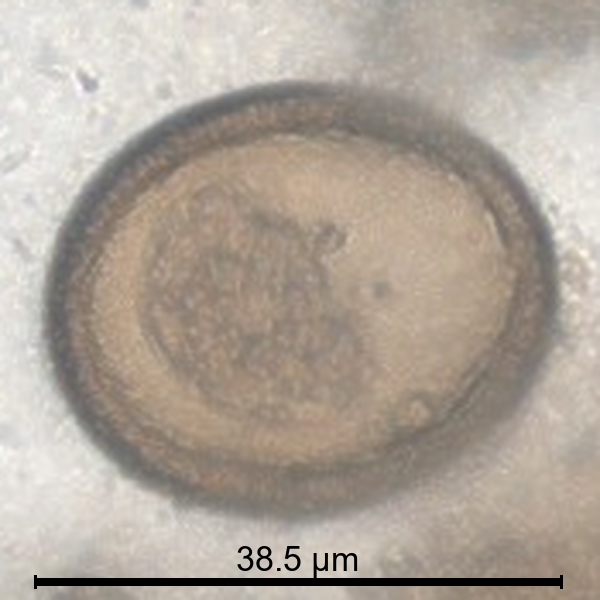 | 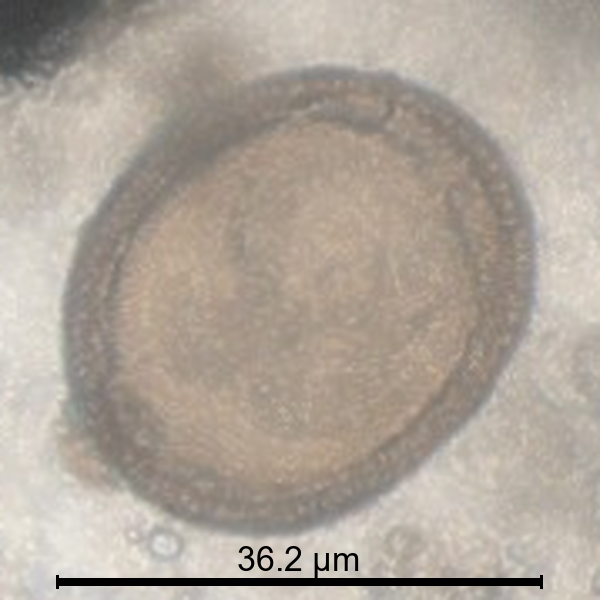 |
| --- | --- | --- | --- |

Group #3. One object marked as hookworm by one technician:

| 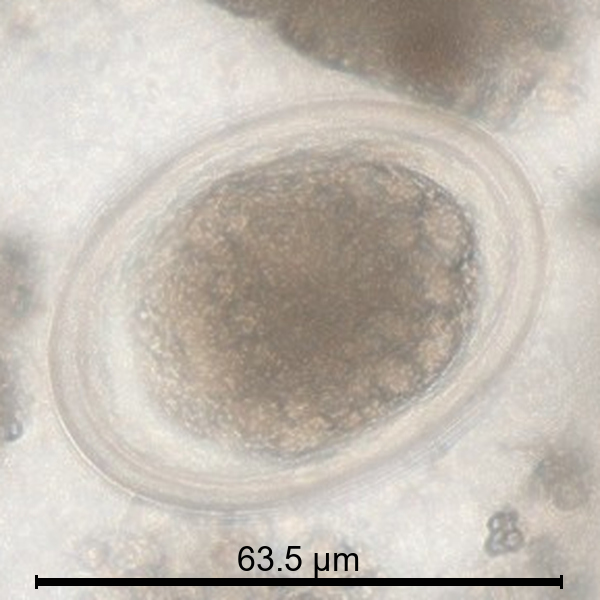 |  |  |  |
| --- | --- | --- | --- |

The technician misclassified the group #3 structure incorrectly as a hookworm egg when reviewing in the AI-assisted mode (without knowing what species the AI model predicted). This misclassification is an example of a failure of the human expert, as the depicted structure clearly is not a hookworm egg because the eggshell is too thick for a hookworm egg. The lack of the mammillated layer (decorticated).might have led to the misclassification.

### *Hymenolepis diminuta*. Slide #37 (3 false negative cases)

The intended results of slide #37 is *Hymenolepis diminuta*.

Group #1 marked as an artifact by three of four technicians and only one technician would study this slide additionally.

| 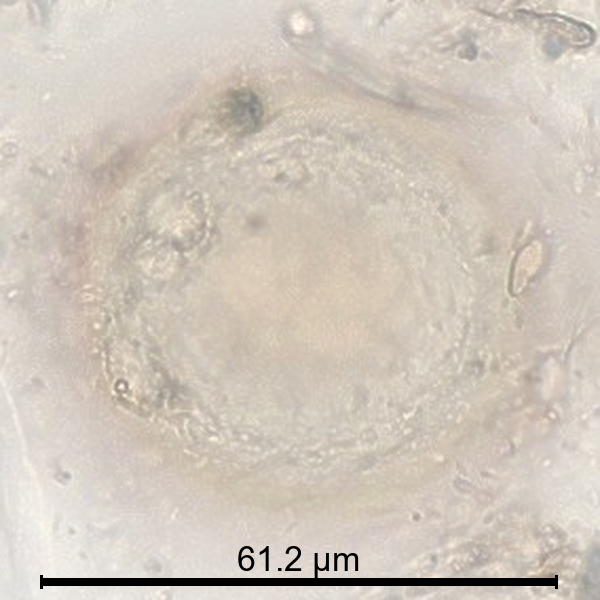 |  |  |  |
| --- | --- | --- | --- |

In this slide only one egg was detected. However, scanning resulted in images with a poor focus in all Z-stacks. The reason for poor focus can be the slide preparation of preserved stool and not concentrated material (e.g. by formol-ether concentration) which usually does not contain so many large structures that can thicken the wet mount specimen.

### *Diphyllobothrium* spp. Slide #131 (1 false positive)

The intended results of this slide are: *Ascaris lumbricoides*, *Hymenolepis nana* and hookworm.

Group #1. Objects marked as *Ascaris lumbricoides* by 4 technicians*:*

| 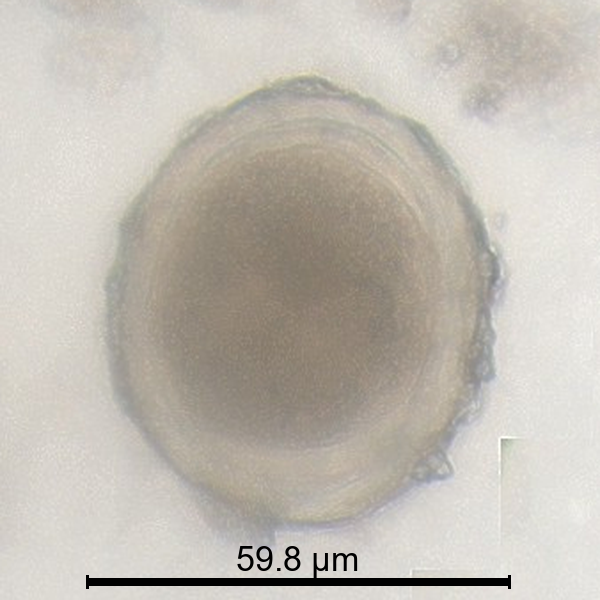 | 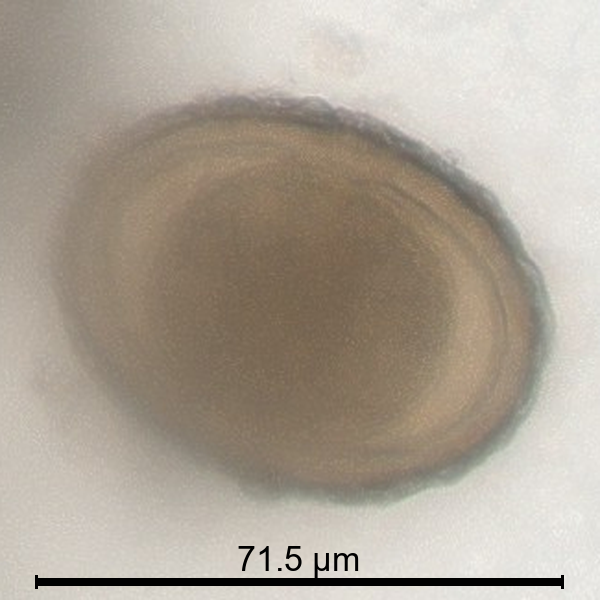 | 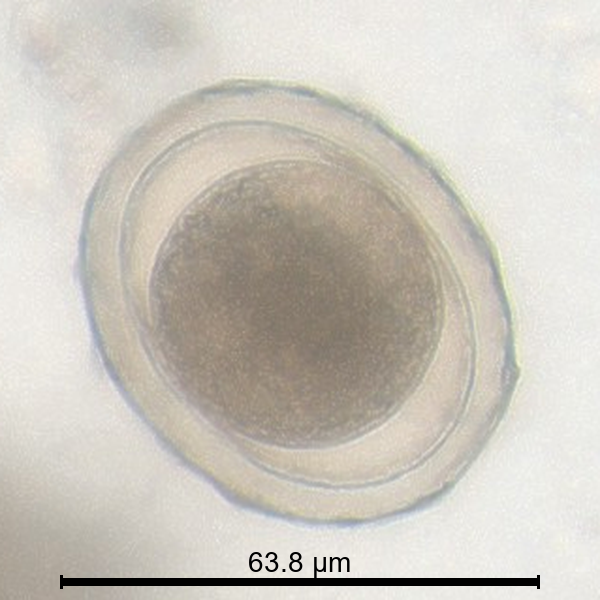 | 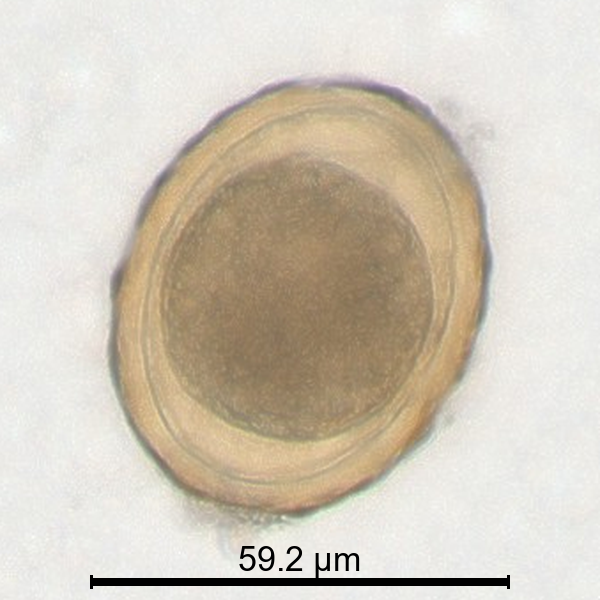 |
| --- | --- | --- | --- |

Group #2. Objects marked as *Hymenolepis nana* by 4 technicians*:*

| 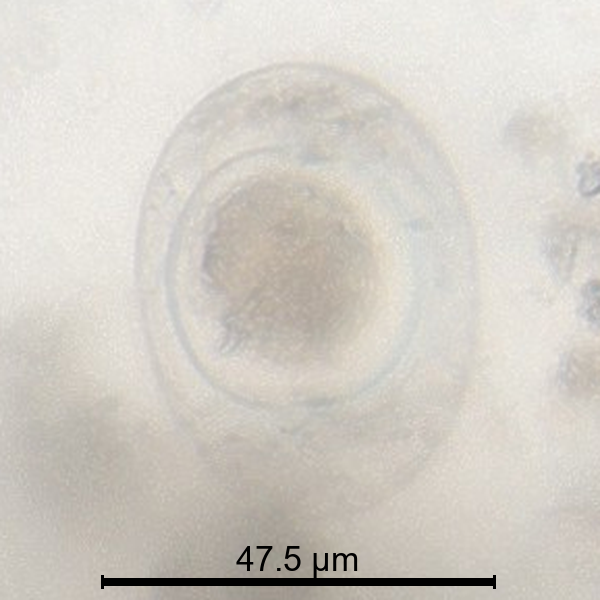 | 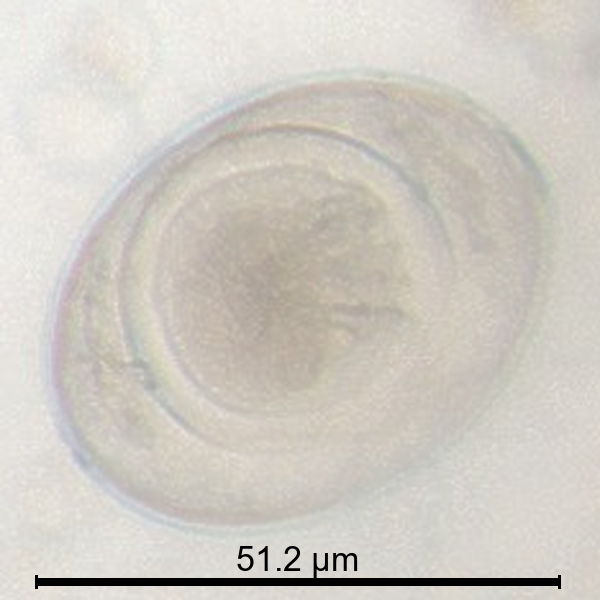 | 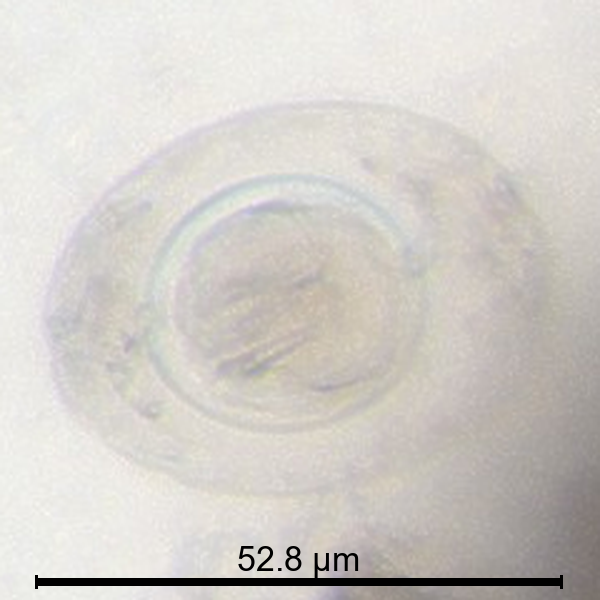 | 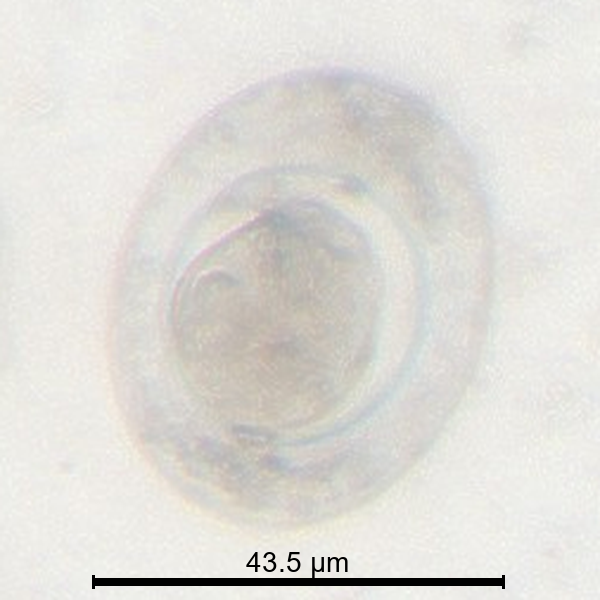 |
| --- | --- | --- | --- |

Group #3. Objects marked as hookworm by 4 technicians*:*

| 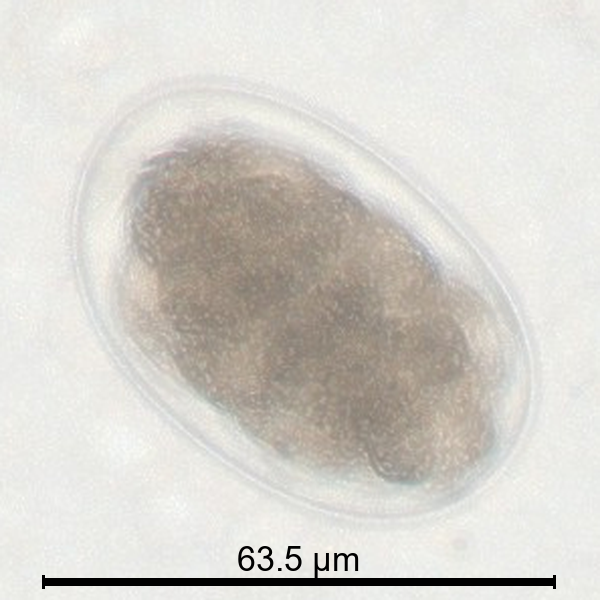 | 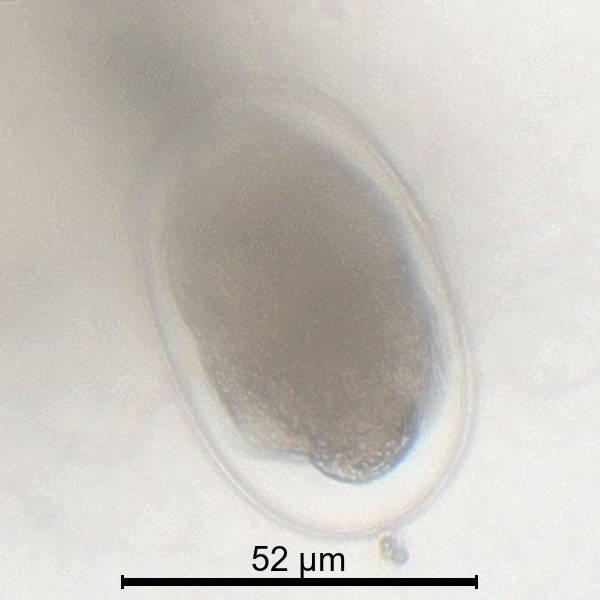 | 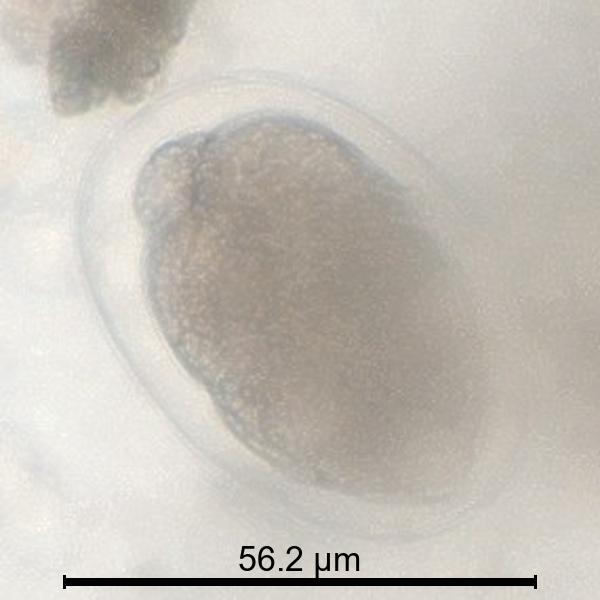 |  |
| --- | --- | --- | --- |

Group #4. First object marked as *Diphyllobothrium* spp. by 1 technician*:*

| 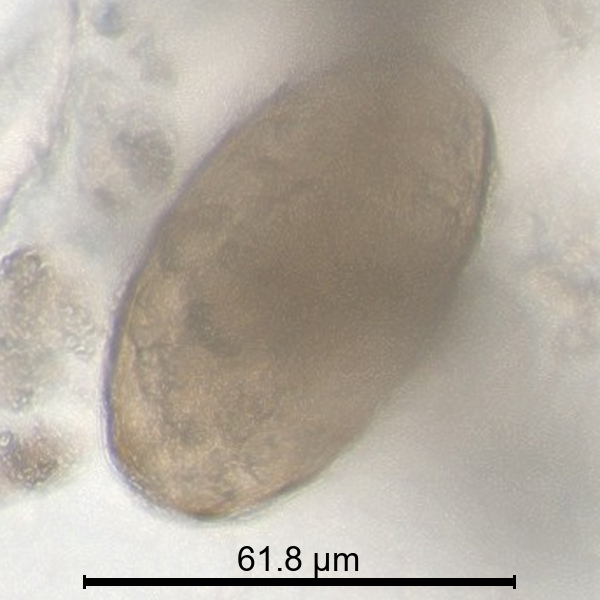 | 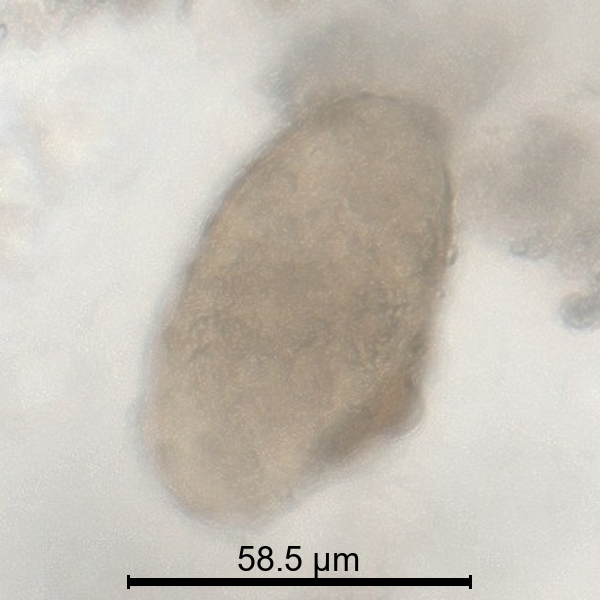 |  |  |
| --- | --- | --- | --- |

*Ascaris lumbricoides* eggs were present as fertilized and unfertilized eggs, and the current model classifies those as two distinct abnormalities. Group #4 contains unfertilized *Ascaris lumbricoides* eggs, where one object was misclassified as *Diphyllobothrium* spp.. This is again a human expert failure, as the helminth eggs in abnormality group 4 are clearly not *Diphyllobothrium* spp. eggs because an operculum is lacking, the eggshell is too thick and bumpy (mammillated).

### *Schistosoma japonicum*. Slide #894 (1 false positive)

The intended results of this slide is: *Schistosoma mansoni*.

Group #1. Objects marked as *Schistosoma mansoni by 4 technicians:*

| 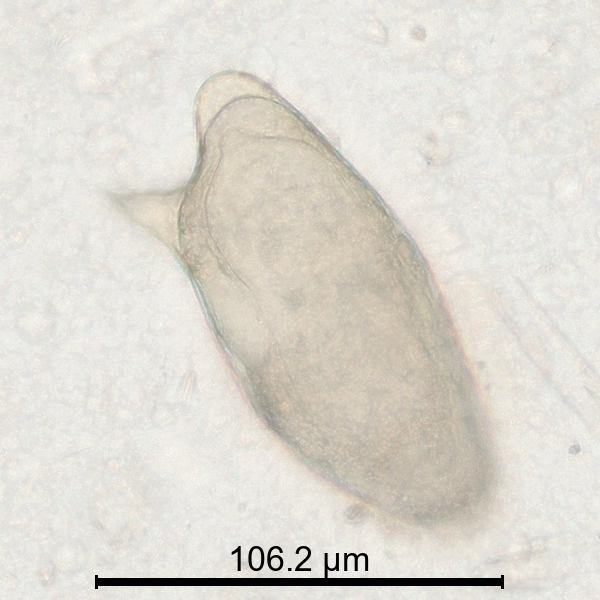 | 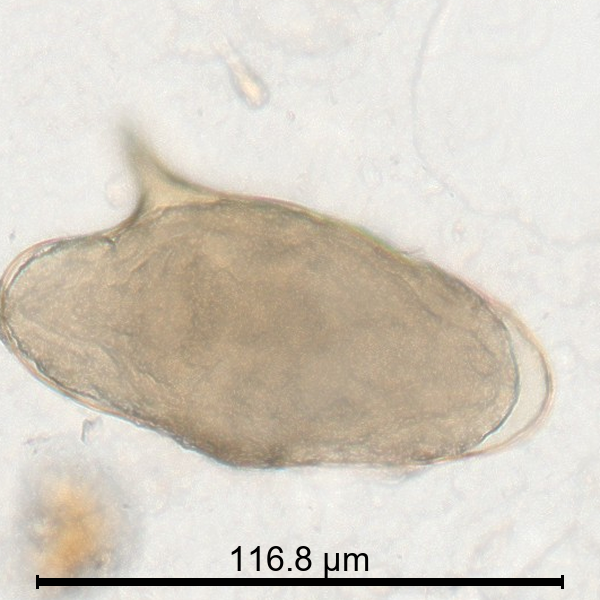 | 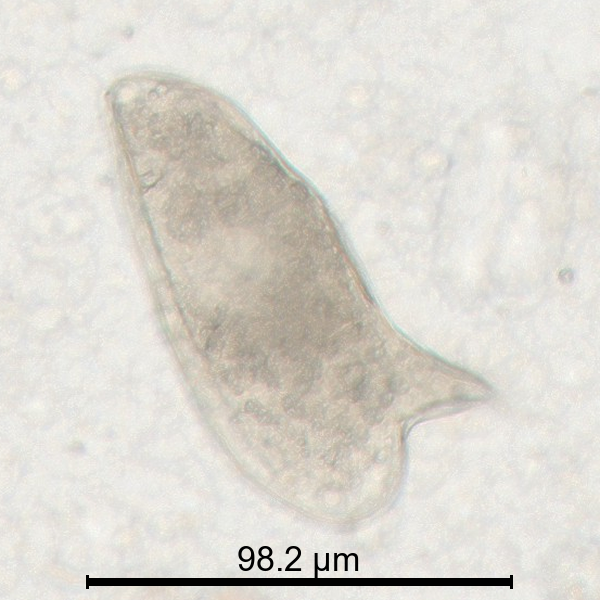 | 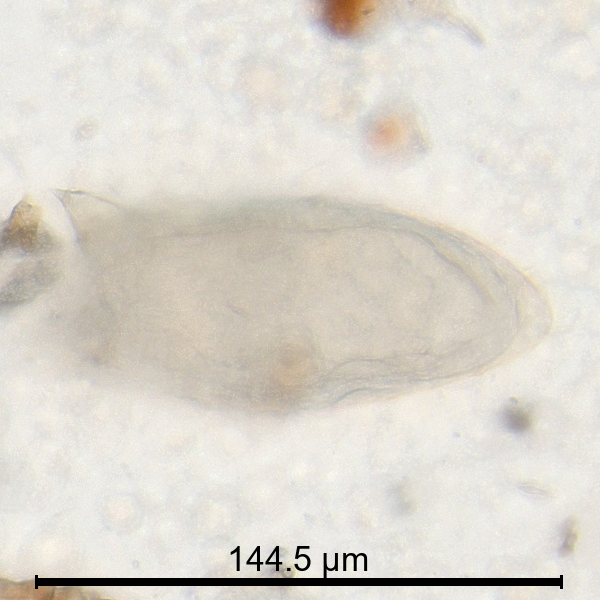 |
| --- | --- | --- | --- |

But additionally, one object from the same group (last image) was marked as *Schistosoma japonicum* by one technician.

Most likely this is an administrative error. The object clearly is a *Schistosoma mansoni* egg, but the next line in the list for selection is *Schistosoma japonicum*, and therefore, the technician probably clicked the wrong item in the list. The chance that the expert technician misinterpreted the results are very low, because the morphology of *S. japonicum* and *S. mansoni* eggs are very different.

The list of species in the user interface presented below.


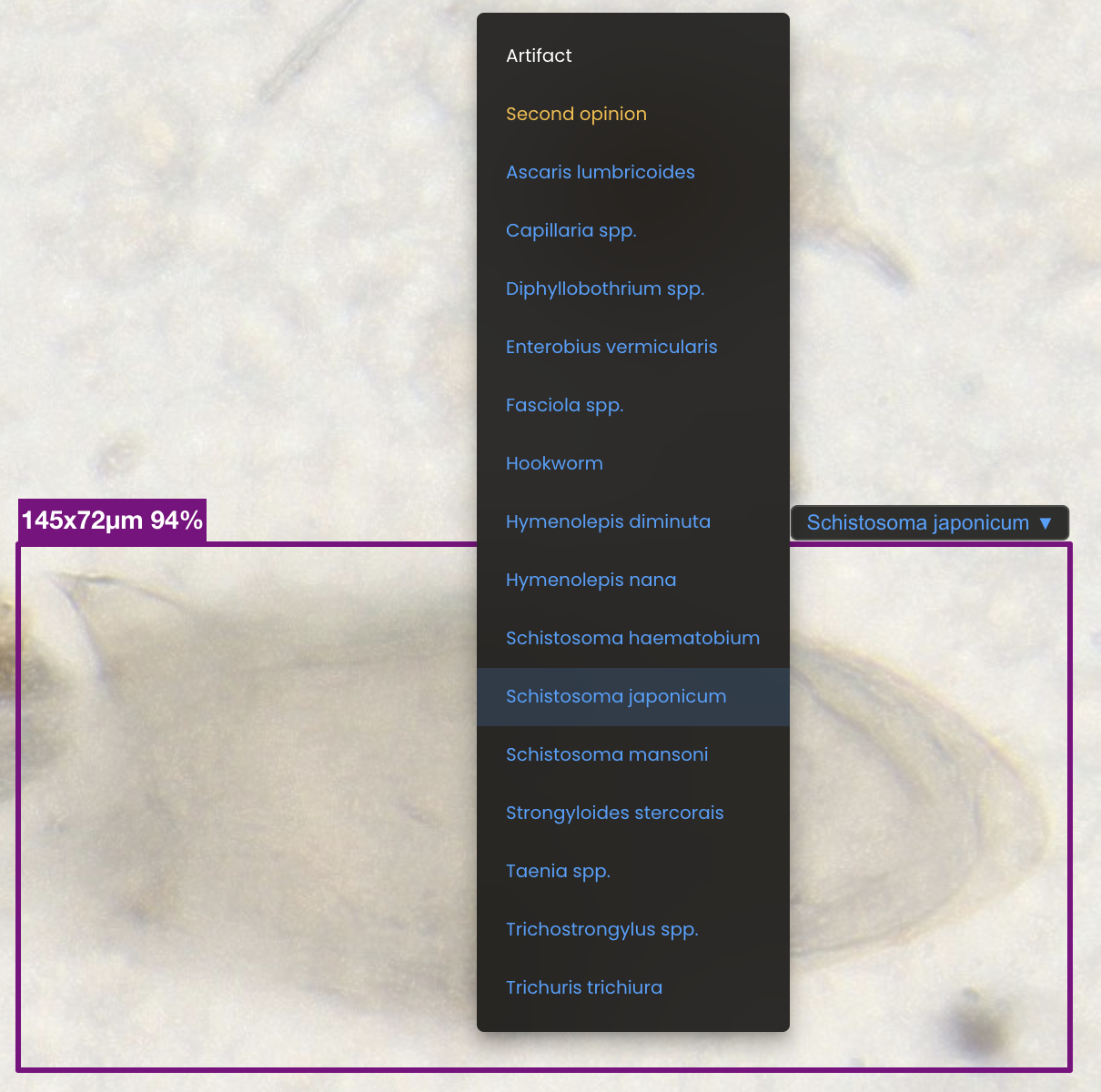


The ParaScout IVD system allows the implementation of a second review process for positive results. Also, once slide analysis is completed in operational settings, technicians review results before final submission. That would prevent administrative errors from occurring in clinical practice.

### *Taenia* spp. Slide #693 (1 false positive)

Intended results were: *Ascaris lumbricoides*, *Schistosoma mansoni*, and *Capillaria* spp..

Group #1. Objects marked as *Ascaris lumbricoides* by 4 technicians*:*

| 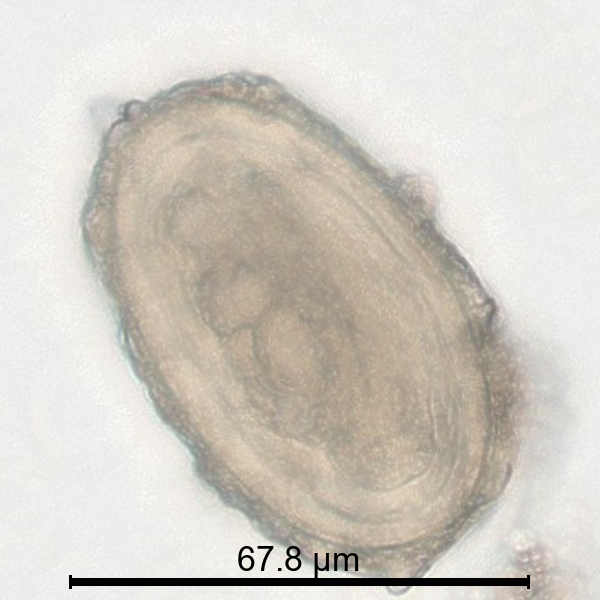 | 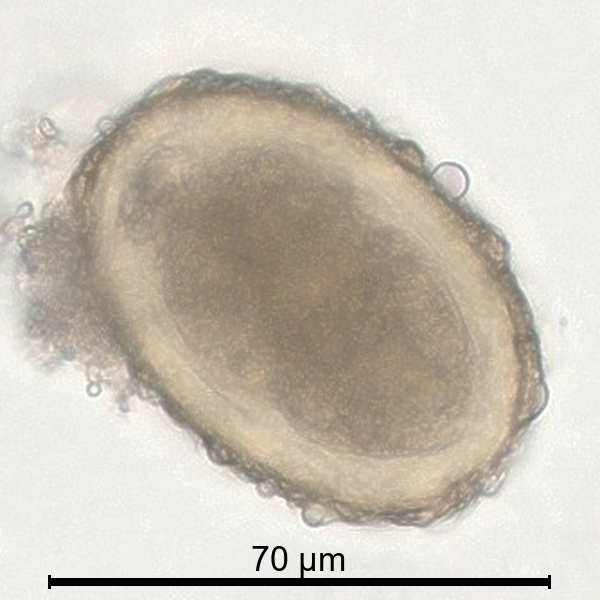 | 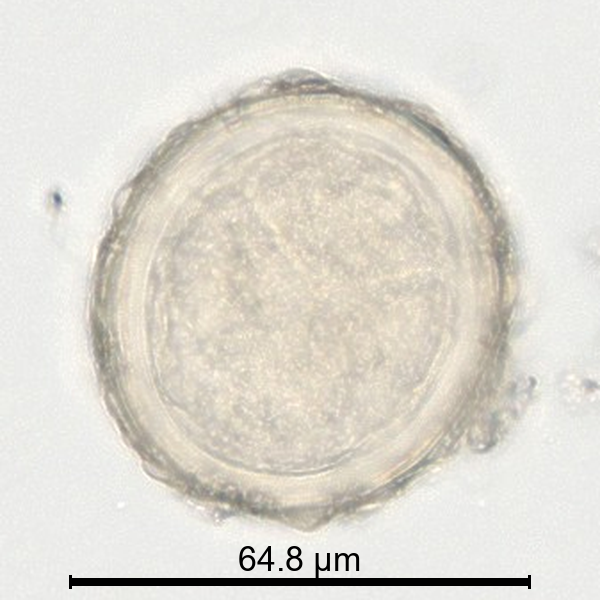 | 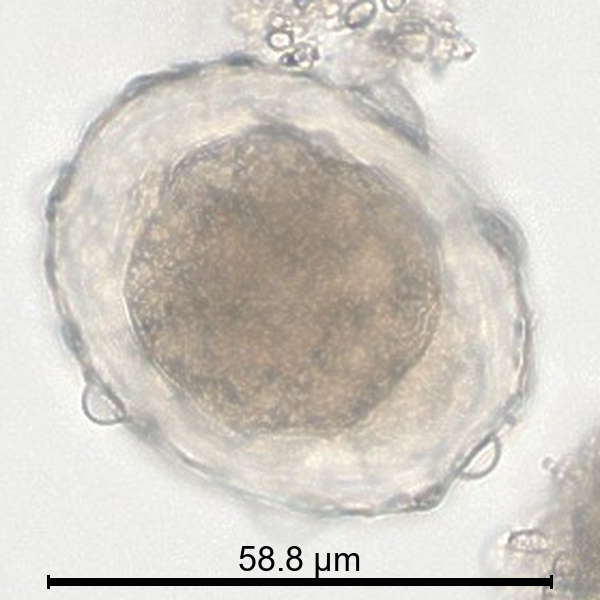 |
| --- | --- | --- | --- |

Group #2. Objects marked as *Schistosoma mansoni* by 4 technicians*:*

| 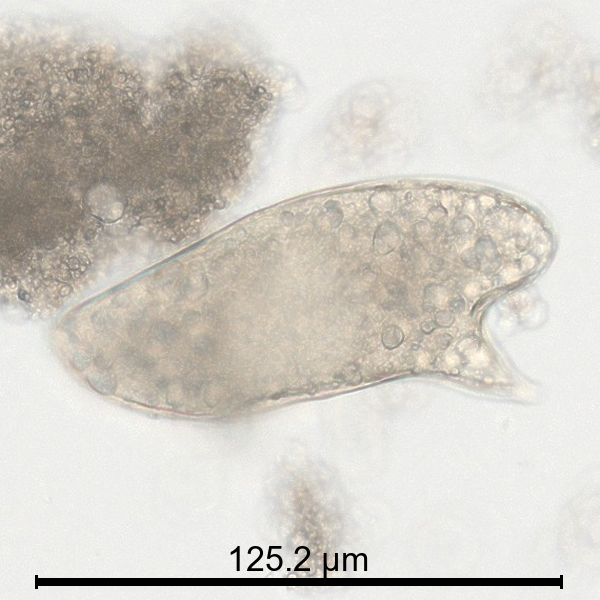 | 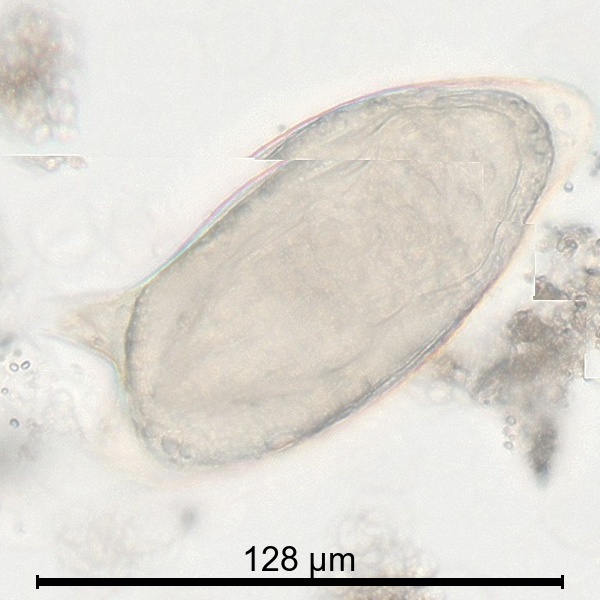 | 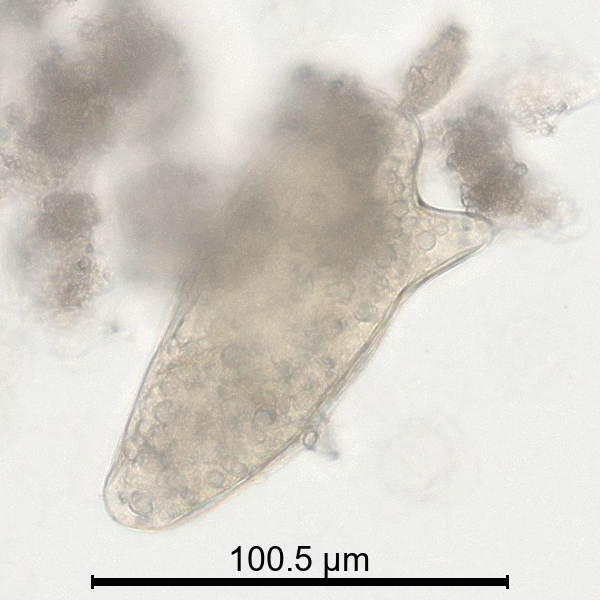 | 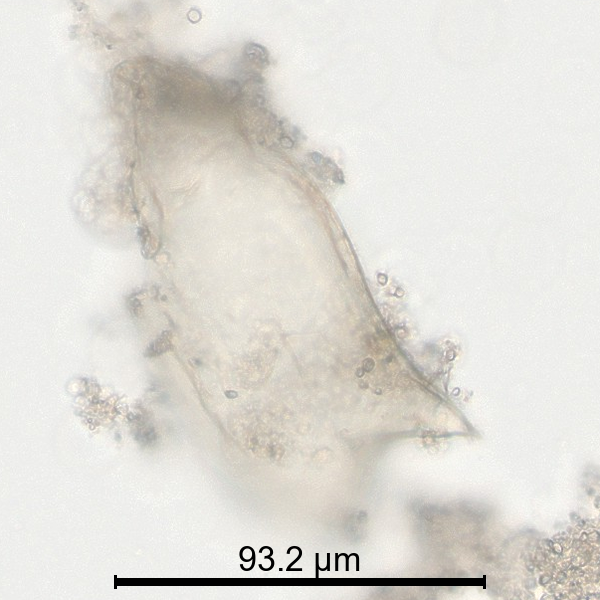 |
| --- | --- | --- | --- |

Group #3. Objects marked as *Capillaria* spp. by 4 technicians*:*

| 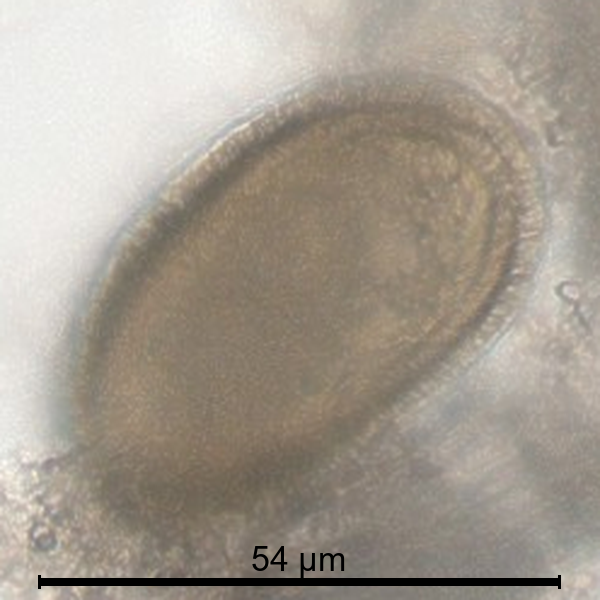 | 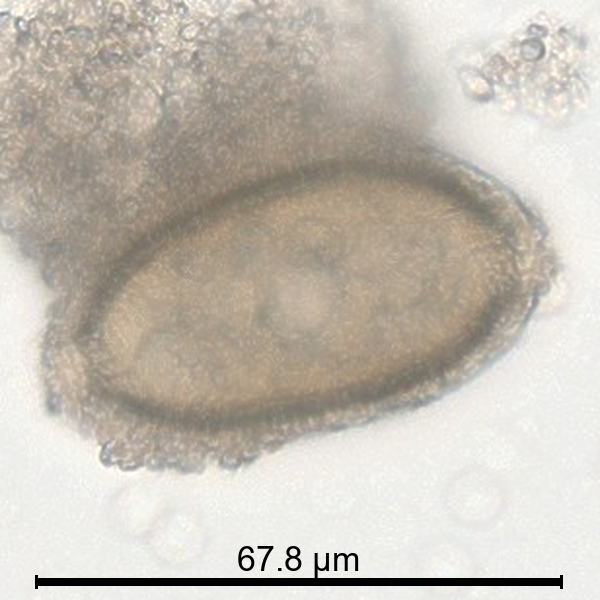 | 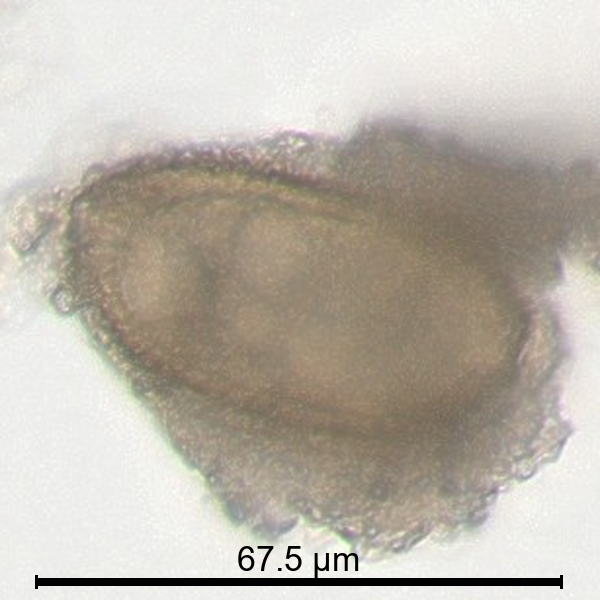 | 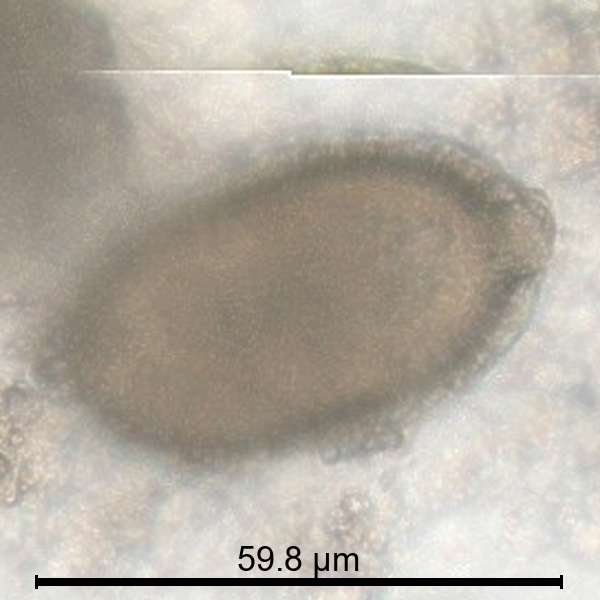 |
| --- | --- | --- | --- |

The ParaScout system detected 6 *Capillaria* spp. eggs and one technician marked two objects as *Taenia* spp. Other technicians marked these objects as Second Opinion, meaning for deeper analysis. Misclassified objects displayed below and again an example of a human expert error. The structures are clearly not *Taenia* spp. eggs, because the structures are too elongated, hooks are lacking and the shell lacks the dark brown color and is not radially striated.

| 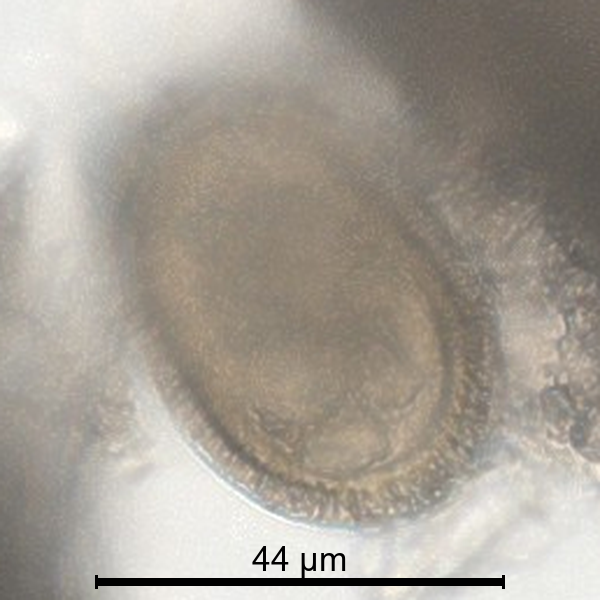 | 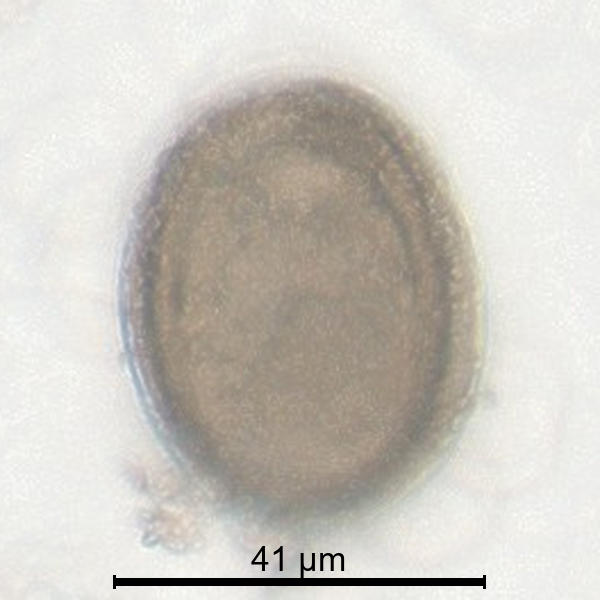 |  |  |
| --- | --- | --- | --- |
