# Supplementary material for: Development and validation of the AI-predictive ParaScout in-vitro diagnostic (IVD) system for the microscopic detection of gastro-intestinal helminths in stool
Source: Emerg Microbes Infect. 2026 Jul 1;15(1):2698240. doi: 10.1080/22221751.2026.2698240 (PMC13366647; doi:10.1080/22221751.2026.2698240)
Supplement: Revised Supplementary Material 8 Determination of final result efficiency of ParaScout.docx [file TEMI_A_2698240_SM7947.docx]

**Supplementary Material 8**

*Determination of final result efficiency of ParaScout*

In some cases the selected structures are not sufficiently clear for experts to confirm the selected structure as a parasite or an artefact. In such a case it was marked as “second opinion”, which means it does need further examination (either digital by 2nd technician or by manual microscopic examination of the specimen).

The ParaScout IVD system groups the selected structures based on the helminth species for which the highest probability score was awarded. However, ParaScout presents the selected structures in blinded abnormality groups to the expert technician, so without showing the suggested helminth species. If one or more objects were correctly identified as the intended parasite, that helminth infection was confirmed and examination for that helminth species was completed. We have analyzed for how many helminth species classes in the reviewed slides, the expert technician selected “second opinion” as the final result, because in such cases the technician could not come to a final conclusion on the presence or absence of that helminth species. From these numbers the efficiency of the AI-predictive ParaScout system could be calculated.

In total 63 helminth species were present in the 50 stool specimens and 4 technicians examined the digital images of the selected structures in abnormality groups in order to confirm the presence of a certain helminth species, define a selected structure as an artifact or to conclude that interpretation was not possible with certainty and that manual examination was required. Table supplementary material 6 shows the efficiency at which a final conclusion could be drawn by the AI-predictive mode of ParaScout.

Table supplementary material 6: Efficiency of the AI-predictive ParaScout IVD system for the detection and identification of helminths in stool

| Observer | Confirmed identification result | No conclusion, needs manual examination | Total | Efficiency  (% confirmed identification of total) |
| --- | --- | --- | --- | --- |
| 1 | 60 | 3 | 63 | 95.2 |
| 2 | 53 | 10 | 63 | 84.1 |
| 3 | 54 | 9 | 63 | 85.7 |
| 4 | 58 | 5 | 63 | 92.1 |
| Total | 225 | 27 | 252 | 89.3 |

For circa 10% of identified parasite classes the ParaScout system could not provide sufficiently clear images to identify enough morphological characteristics for a direct conclusion to confirm or to reject the presence of a specific helminth species. Therefore, the efficiency varied substantially between technicians (range 85 to 95%).

## **Additional analysis of True Negative slides**

In addition to the main study described in the manuscript, 22 sediments of formol-ether concentrates of clinical samples were also examined by the ParaScout system. In these stool samples no helminths were detected by routine manual examination for clinical purposes. Parascout examination of these specimens resulted in the detection of only a small number of structures with a predictive value above the threshold of 0.6 to be suspected to be a helminth. On average 0.77 structures were detected per 22x22 mm slide.

During the analysis of 22 True Negative slides, the model had detected 17 objects in the examined slides and these cases are described below.

| **Slide** | **Total FPs** | **Unique Species** | **Species Breakdown** |
| --- | --- | --- | --- |
| TN1 | **4** | 2 | *Strongyloides stercoralis*: 3, *Hymenolepis nana*: 1 |
| TN2 | **3** | 2 | *Strongyloides stercoralis*: 2, *Hymenolepis nana*: 1 |
| TN3 | **2** | 2 | *Taenia* spp.: 1, *Ascaris lumbricoides*: 1 |
| TN4 | **2** | 2 | *Trichuris trichiura*: 1, *Hymenolepis diminuta*: 1 |
| TN5 | **1** | 1 | *Strongyloides stercoralis*: 1 |
| TN6 | **1** | 1 | *Taenia* spp.: 1 |
| TN7 | **1** | 1 | *Strongyloides stercoralis*: 1 |
| TN8 | **1** | 1 | *Enterobius vermicularis*: 1 |
| TN9 | **1** | 1 | *Strongyloides stercoralis*: 1 |
| TN10 | **1** | 1 | *Schistosoma mansoni*: 1 |
| TN11 | **0** | 0 |  |
| TN12 | **0** | 0 |  |
| TN13 | **0** | 0 |  |
| TN14 | **0** | 0 |  |
| TN15 | **0** | 0 |  |
| TN16 | **0** | 0 |  |
| TN17 | **0** | 0 |  |
| TN18 | **0** | 0 |  |
| TN19 | **0** | 0 |  |
| TN20 | **0** | 0 |  |
| TN21 | **0** | 0 |  |
| TN22 | **0** | 0 |  |
| **TOTAL** | **17** | **12** |  |

**TN1.** In this case 2 distinct variants of misclassification were identified. First - it's unknown why but these dotted patterns were misclassified as *Strongyloides stercoralis*. Notably this issue has been resolved on the newer models.

| 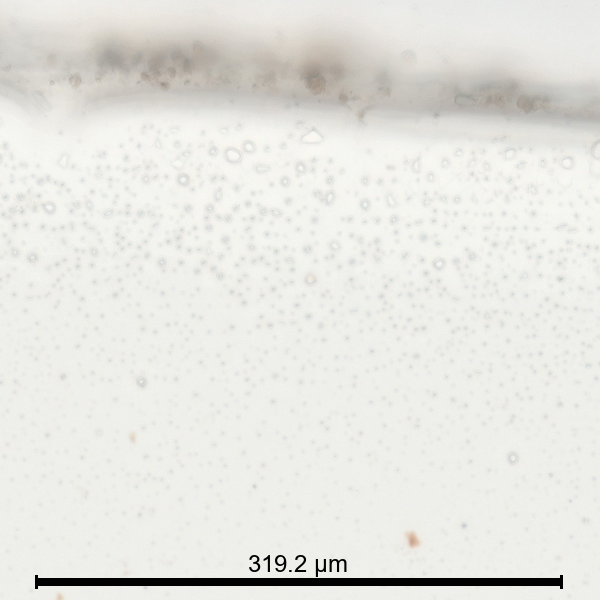 |
| --- |

Second, this bubble was misclassified as *Hymenopolis nana* - probably due to its size, color and the outer edges of the bubble looking similar to the egg walls. Because no internal structures are present, this is clearly a misidentification and therefore it is likely to be determined as an artefact by expert reviewing of the identified structure.

| 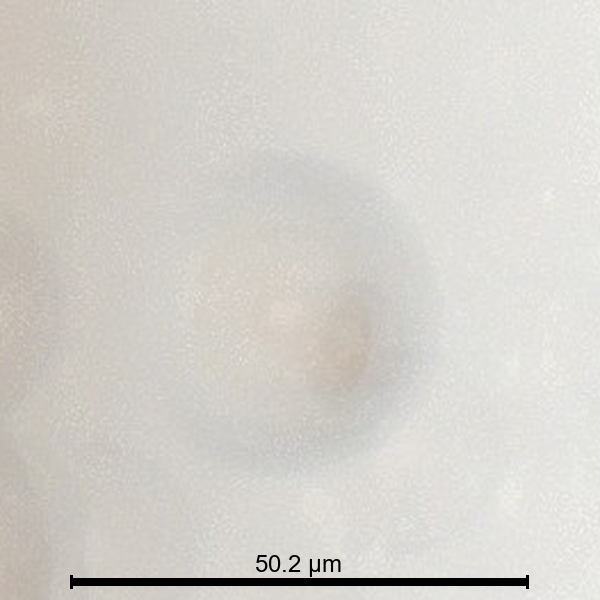 |
| --- |

**TN2.** Just like in the previous case 2 types of misclassifications were detected: the dotted pattern in the left panel below was classified as *Strongyloides stercoralis* and the bubble-like objects were classified as *Hymenopolis nana.* Again both identified structures lack critical morphological features of the supposed helminth species, these are clear misidentifications and therefore it is likely that those will be determined as artefacts by expert reviewing of the identified structure.

| 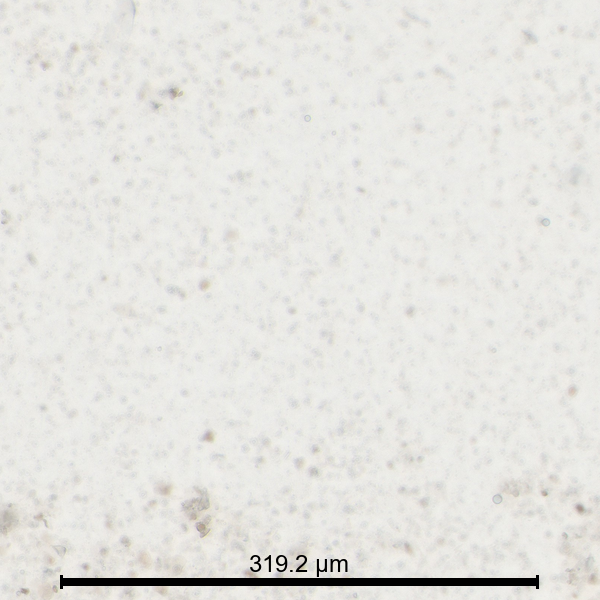 | 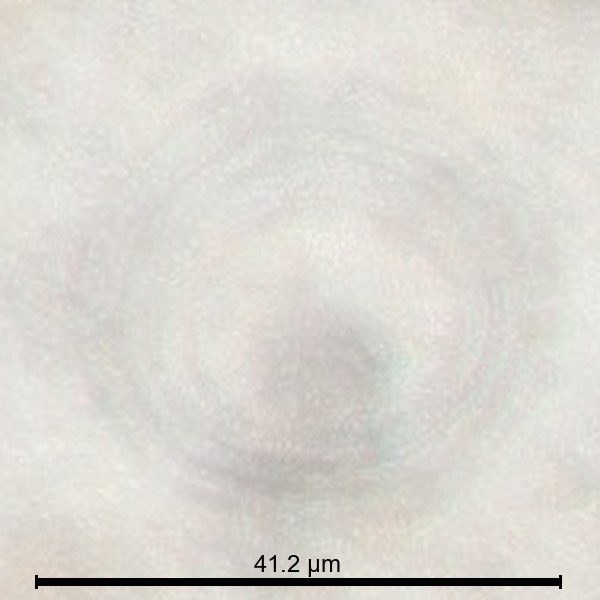 |
| --- | --- |

**TN3.** In this slide both False Positive identifications (*Ascaris* species and *Taenia* species, in left and right panel below respectively) can be attributed to floating debris in the slide. Both identified structures share color, size and general shape to the supposed helminth eggs. Again both identified structures are clear misidentifications as a continuous and regular eggshell is lacking. Hence it is likely that those will be determined as artefacts by expert reviewing of the identified structure.

| 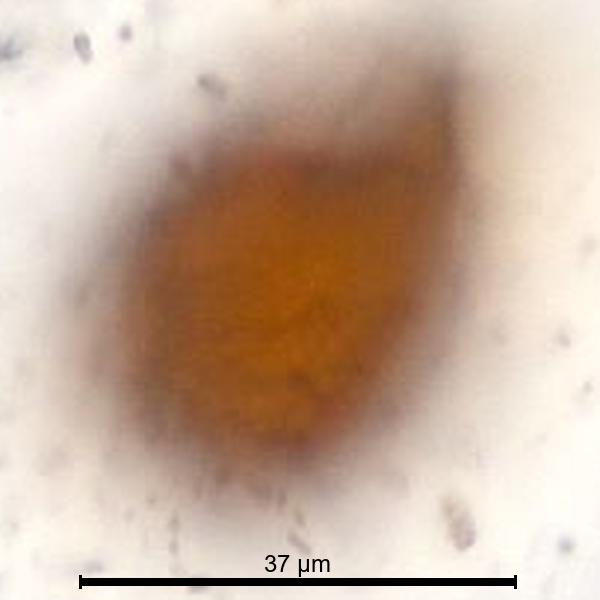 | 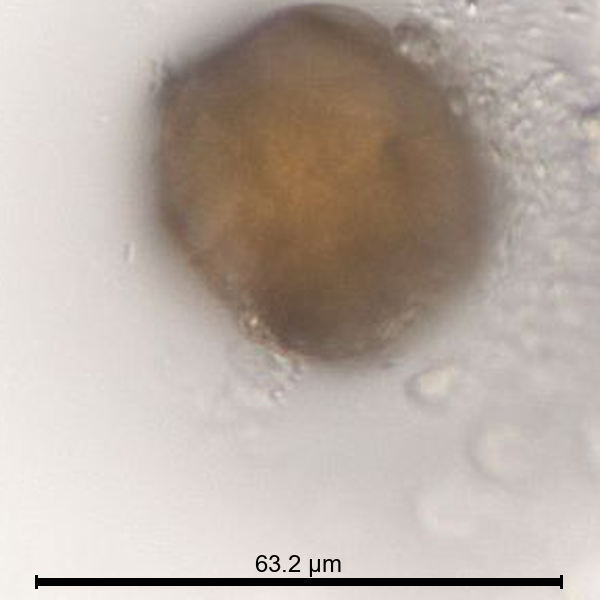 |
| --- | --- |

**TN4.** Same behaviour as in TN3 - parts of debris present in the slide were misclassified as the *Trichuris trichiura* or *Hymenolepis diminuta* (in left and right panel below, respectively). These structures are clearly not helminth eggs, as a proper eggshell is lacking.

| 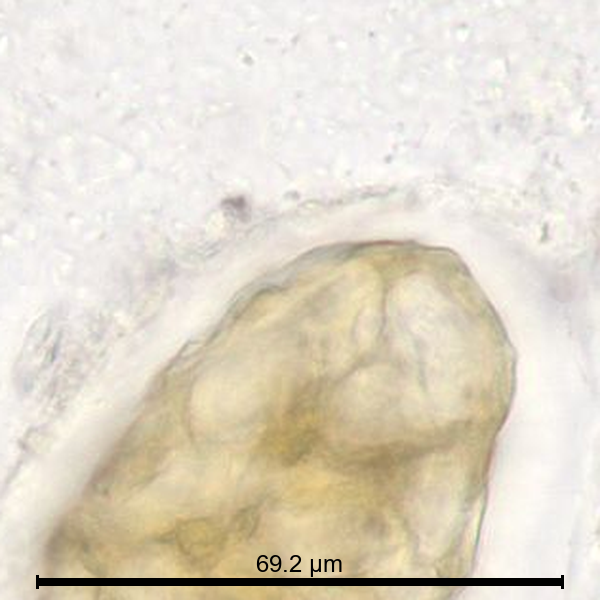 | 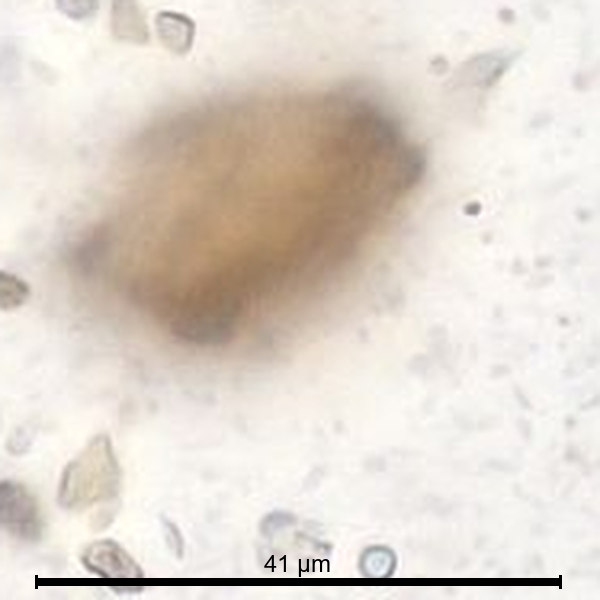 |
| --- | --- |

**TN5.** In this case the edge of a cover glass that was misidentified as *Strongyloides stercoralis,* and therefore, also are clear misidentification that will be declined by expert reviewing.

| 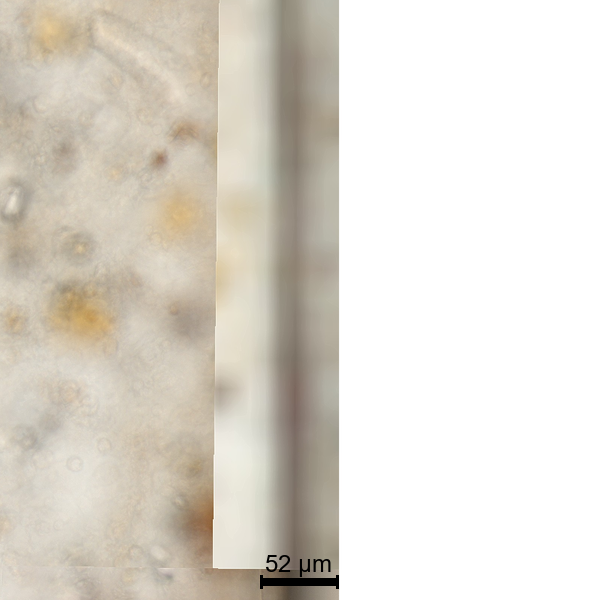 |
| --- |

**TN6.** Here the False detection was caused due to a combination of 2 issues. First - this debris has a form which is vaguely similar to a *Taenia* egg, and due to a stitching artifact it became blurry, which confused the model even further. Again this identified structure is a clear misidentification as critical morphological features are lacking. Hence it is likely that this structure will be determined as an artefact by expert reviewing of the identified structure.

| **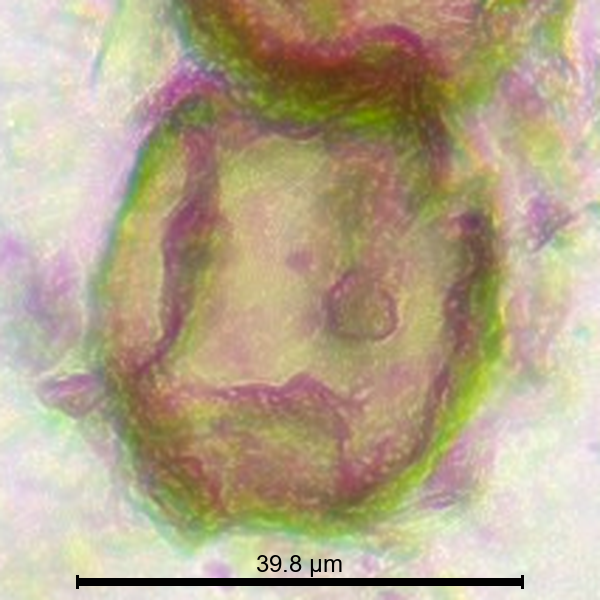** |
| --- |

**TN7.** Here an unknown object was misclassified as a *Strongyloides stercoralis* larvae, due to it being genuinely similar to a *Strongyloides stercoralis* larvae. It’s worth noting that blurriness on one of the layers likely contributed to the identification, as on a layer with better clarity the object wasn't detected. Again this identified structure is a clear misidentification as critical morphological features are lacking. Hence it is likely that this structure will be determined as an artefact by expert reviewing of the identified structure.

| **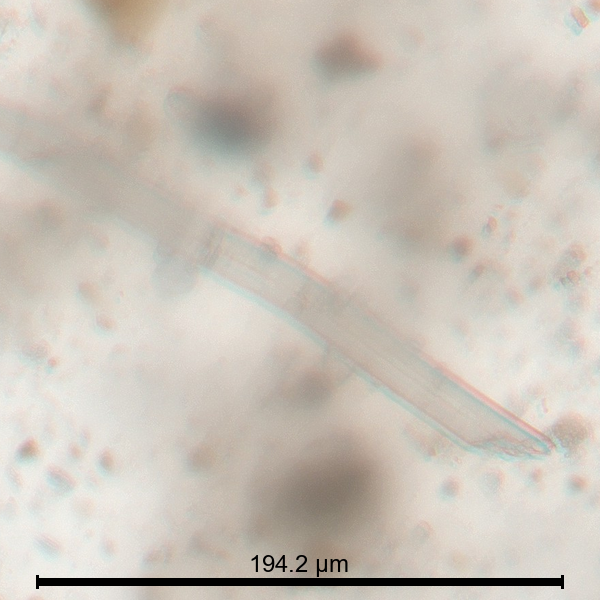** |  |
| --- | --- |

**TN8.** Here an unknown artifact was classified as *Enterobius vermicularis*. Notably on the newer models this issue has been resolved. Again this identified structure is a clear misidentification as critical morphological features are lacking. Hence it is likely that this structure will be determined as an artefact by expert reviewing of the identified structure.

| 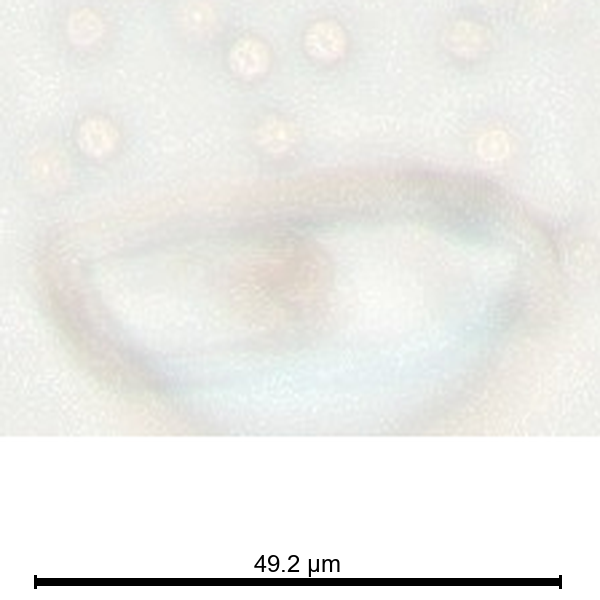 |
| --- |

**TN9.** On this slide a piece of unknown debris was identified as a possible *Strongyloides stercoralis* larvae, as it shares some features with this helminth. However, this identified structure is also a clear misidentification as critical morphological features are lacking. Hence it is likely that this structure will be determined as an artefact by expert reviewing of the identified structure.

| 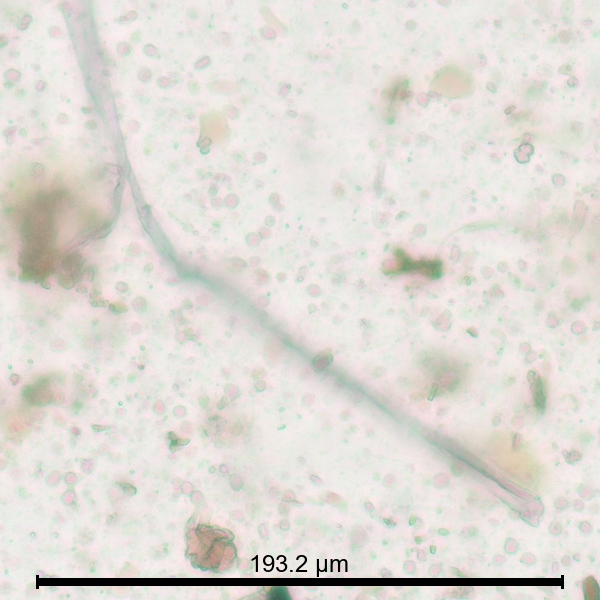 |
| --- |

**TN10.** In this slide we have an imaging artifact misclassified as a *Schistosoma mansoni* for an unknown reason. Notably this behaviour was fixed in later model versions.

| 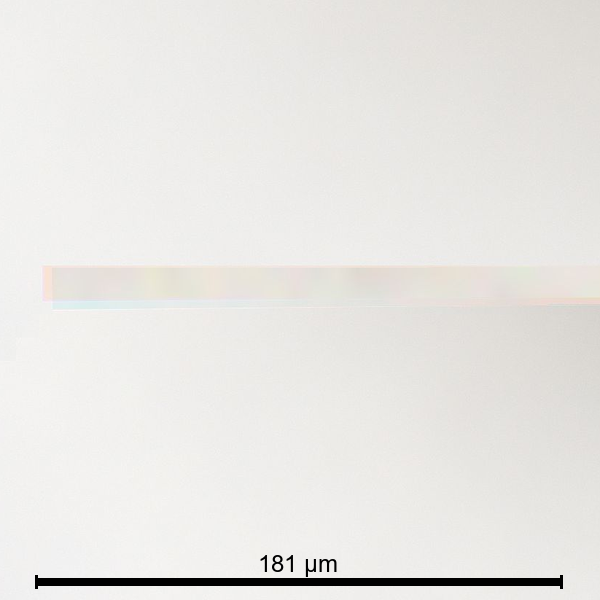 |
| --- |
