# Supplementary material for: Development and validation of the AI-predictive ParaScout in-vitro diagnostic (IVD) system for the microscopic detection of gastro-intestinal helminths in stool
Source: Emerg Microbes Infect. 2026 Jul 1;15(1):2698240. doi: 10.1080/22221751.2026.2698240 (PMC13366647; doi:10.1080/22221751.2026.2698240)
Supplement: Feoktistov et al Suppl data 3 List of helminth species.pdf [file TEMI_A_2698240_SM7943.pdf]

Supplementary Material 3

List of the 15 helminth species for which the ParaScout algorithm was trained at the time the performance evaluation experiment was performed:

a. *Ascaris* species
b. *Capillaria* species
c. *Diphyllobothrium* spp.
d. *Enterobius vermicularis*
e. *Fasciola* species
f. Hookworm species
g. *Hymenolepis diminuta*
h. *Hymenolepis nana*
i. *Schistosoma haematobium*
j. *Schistosoma japonicum*
k. *Schistosoma mansoni*
l. *Strongyloides stercoralis*
m. *Taenia* species
n. *Trichostrongylus* species
o. *Trichuris trichiura*
