# Supplementary material for: Development and validation of the AI-predictive ParaScout in-vitro diagnostic (IVD) system for the microscopic detection of gastro-intestinal helminths in stool
Source: Emerg Microbes Infect. 2026 Jul 1;15(1):2698240. doi: 10.1080/22221751.2026.2698240 (PMC13366647; doi:10.1080/22221751.2026.2698240)
Supplement: Feoktistov et al Suppl data 6 performance of ParaScout.pdf [file TEMI_A_2698240_SM7942.pdf]

Supplementary Material 6

Performance of fully automated ParaScout examination.
In total 50 stool specimens were examined by the ParaScout IVD system for the presence or absence of 15 helminth species, which produced in total 50*15=750 results. Because all stool samples were validated for the presence and absence of helminth species, the intended results could be used to classify the results of the ParaScout IVD system in its fully automated mode as either true positive (TP) false positive (FP), true negative (TN) or false negative (FN). This analysis was performed with every confidence threshold between 0.25 and 1.

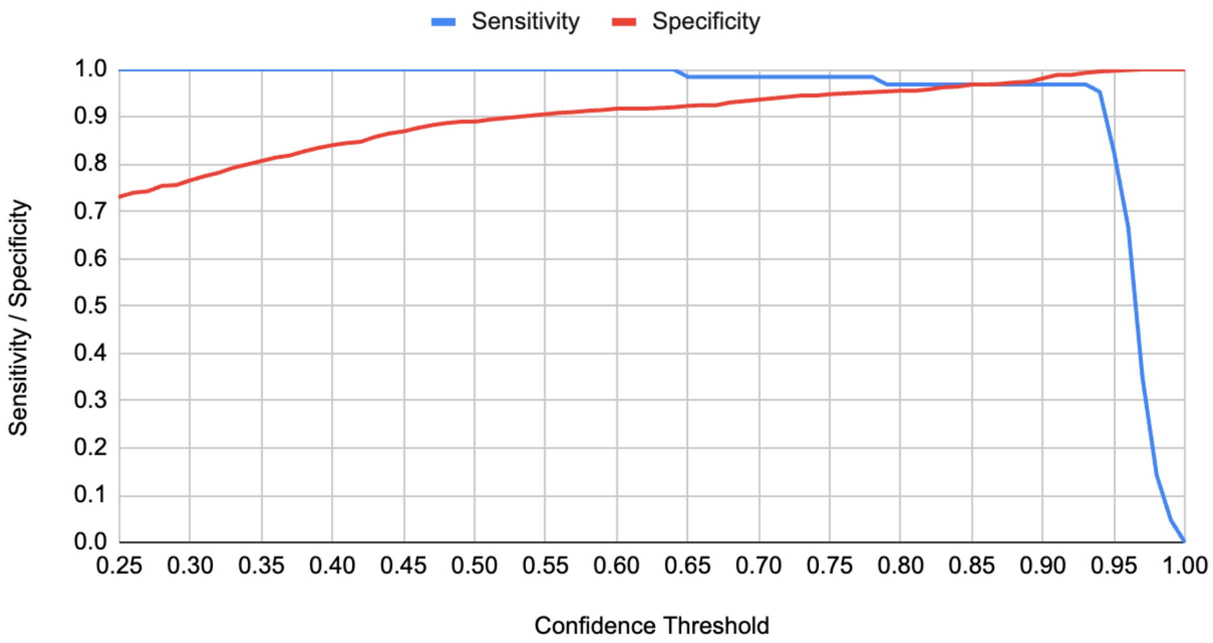

Supplementary material 6, figure 1. The sensitivity (blue) and specificity (red) of the fully automated ParaScout IVD system as a variable of the confidence threshold.

At 0.6 confidence threshold the sensitivity of the ParaScout IVD system still has a sensitivity of 100% (no false negative results), but the specificity is only 91.70%. By increasing the confidence threshold the specificity increases to 100%, but the sensitivity then decreases. From this information a Receiver Operating Curve (ROC) was prepared.

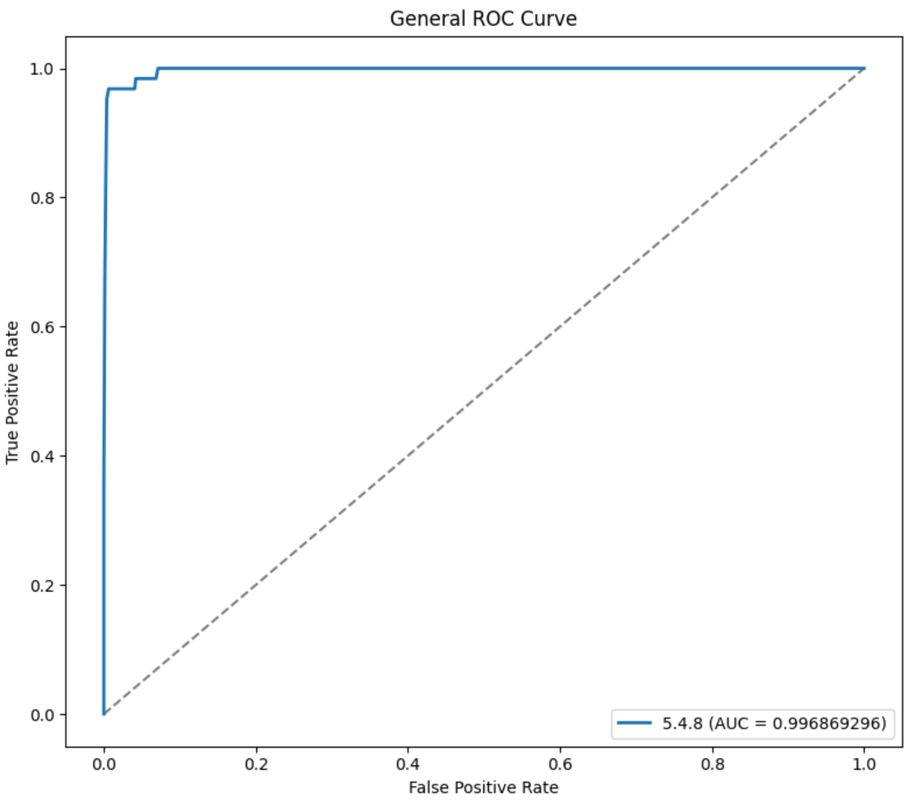

Supplementary material 6, figure 2. Receiver Operating Curve (ROC) of the fully automated ParaScout IVD system with an area under curve (AUC) of 0.997.
